# Differential Proteome Analysis of Chikungunya Virus Infection on Host Cells

**DOI:** 10.1371/journal.pone.0061444

**Published:** 2013-04-10

**Authors:** Christina Li-Ping Thio, Rohana Yusof, Puteri Shafinaz Akmar Abdul-Rahman, Saiful Anuar Karsani

**Affiliations:** 1 Institute of Biological Sciences, Faculty of Science, University of Malaya, Kuala Lumpur, Malaysia; 2 Department of Molecular Medicine, Faculty of Medicine, University of Malaya, Kuala Lumpur, Malaysia; 3 Medical Biotechnology Laboratory, Faculty of Medicine, University of Malaya Centre for Proteomics Research (UMCPR), University of Malaya, Kuala Lumpur, Malaysia; 4 Drug Design and Development Research Group (DDDRG), University of Malaya, Kuala Lumpur, Malaysia; University of Edinburgh, United Kingdom

## Abstract

**Background:**

Chikungunya virus (CHIKV) is an emerging mosquito-borne alphavirus that has caused multiple unprecedented and re-emerging outbreaks in both tropical and temperate countries. Despite ongoing research efforts, the underlying factors involved in facilitating CHIKV replication during early infection remains ill-characterized. The present study serves to identify host proteins modulated in response to early CHIKV infection using a proteomics approach.

**Methodology and Principal Findings:**

The whole cell proteome profiles of CHIKV-infected and mock control WRL-68 cells were compared and analyzed using two-dimensional gel electrophoresis (2-DGE). Fifty-three spots were found to be differentially modulated and 50 were successfully identified by MALDI-TOF/TOF. Eight were significantly up-regulated and 42 were down-regulated. The mRNA expressions of 15 genes were also found to correlate with the corresponding protein expression. STRING network analysis identified several biological processes to be affected, including mRNA processing, translation, energy production and cellular metabolism, ubiquitin-proteasome pathway (UPP) and cell cycle regulation.

**Conclusion/Significance:**

This study constitutes a first attempt to investigate alteration of the host cellular proteome during early CHIKV infection. Our proteomics data showed that during early infection, CHIKV affected the expression of proteins that are involved in mRNA processing, host metabolic machinery, UPP, and cyclin-dependent kinase 1 (CDK1) regulation (in favour of virus survival, replication and transmission). While results from this study complement the proteomics results obtained from previous late host response studies, functional characterization of these proteins is warranted to reinforce our understanding of their roles during early CHIKV infection in humans.

## Introduction

Chikungunya (CHIK) is a long-neglected disease that only recently began to garner attention from the scientific community following devastating outbreaks that struck India and the Indian Ocean Islands from 2004 to 2007. This disease causes substantial morbidity and an estimated death rate of 1∶1,000 [1]. Despite being perceived as a tropical disease, recent CHIK cases and sporadic outbreaks were documented in temperate regions, suggesting that this infectious disease is no longer geographically restricted to tropical countries [2]. In Malaysia, three separate outbreaks have been reported over the past 15 years [3], [4], [5].

The causative agent for CHIK infection is the chikungunya virus (CHIKV), an alphavirus belonging to the family *Togaviridae* [6]. CHIKV is transmitted by the mosquito *Aedes aegypti* and *Aedes albopictus*. CHIKV can be genotypically classified into the East Central South African, West African and Asian genotypes [7]. Upon infection, CHIKV causes an acute illness characterized by the classical triad of symptoms of fever, rash and debilitating arthralgia which can persist for years. However, cases from recent outbreaks saw an increasing occurrence of atypical clinical manifestations such as neurological and cardiovascular complications [8]. As there is currently no effective vaccine or antiviral regimen to combat this disease, treatment is solely palliative. All things considered, it is not surprising that CHIK is now regarded as a potential health problem in need of a solution.

Recent research efforts have focused on understanding the viral tropism and mechanisms associated with the pathogenesis of CHIK infection. *In vitro* studies using a panel of mammalian cell lines showed rapid induction of cytopathic effects and cell death via apoptosis in most adherent cell lines with the exception of blood-derived cell lines [9]. Autophagic process and apoptosis were also recently shown to facilitate CHIKV dissemination [10], [11]. At the molecular level, proteomics studies on CHIKV interaction with vector and mammalian host proteins have unravelled new clues in elucidating the mechanisms involved in viral replication and transmission from vector to host as well as disease progression in host cells [12], [13], [14]. Despite the extensive research, much remains to be discovered to fully comprehend the pathogenesis of CHIKV.

Contrary to the aforementioned proteomics research which investigated the late host response to CHIKV infection [13], our present study aims to identify proteins altered during early infection in the host cells by means of 2-dimensional gel electrophoresis (2-DGE). The global proteome profile of CHIKV-infected WRL-68 cells was compared with uninfected mock control cells to single out differentially expressed spots for mass spectrometric (MS) identification with subsequent Western blot validation, as well as transcript expression analysis. Results showed widespread alteration of proteins involved in several biological processes known to play essential roles in virus replication. While this study provides new insights into CHIKV pathogenesis, functional characterization of these proteins will be required to better understand their roles during early infection.

## Results

### Cytopathogenicity of CHIKV

**Figure 1:**
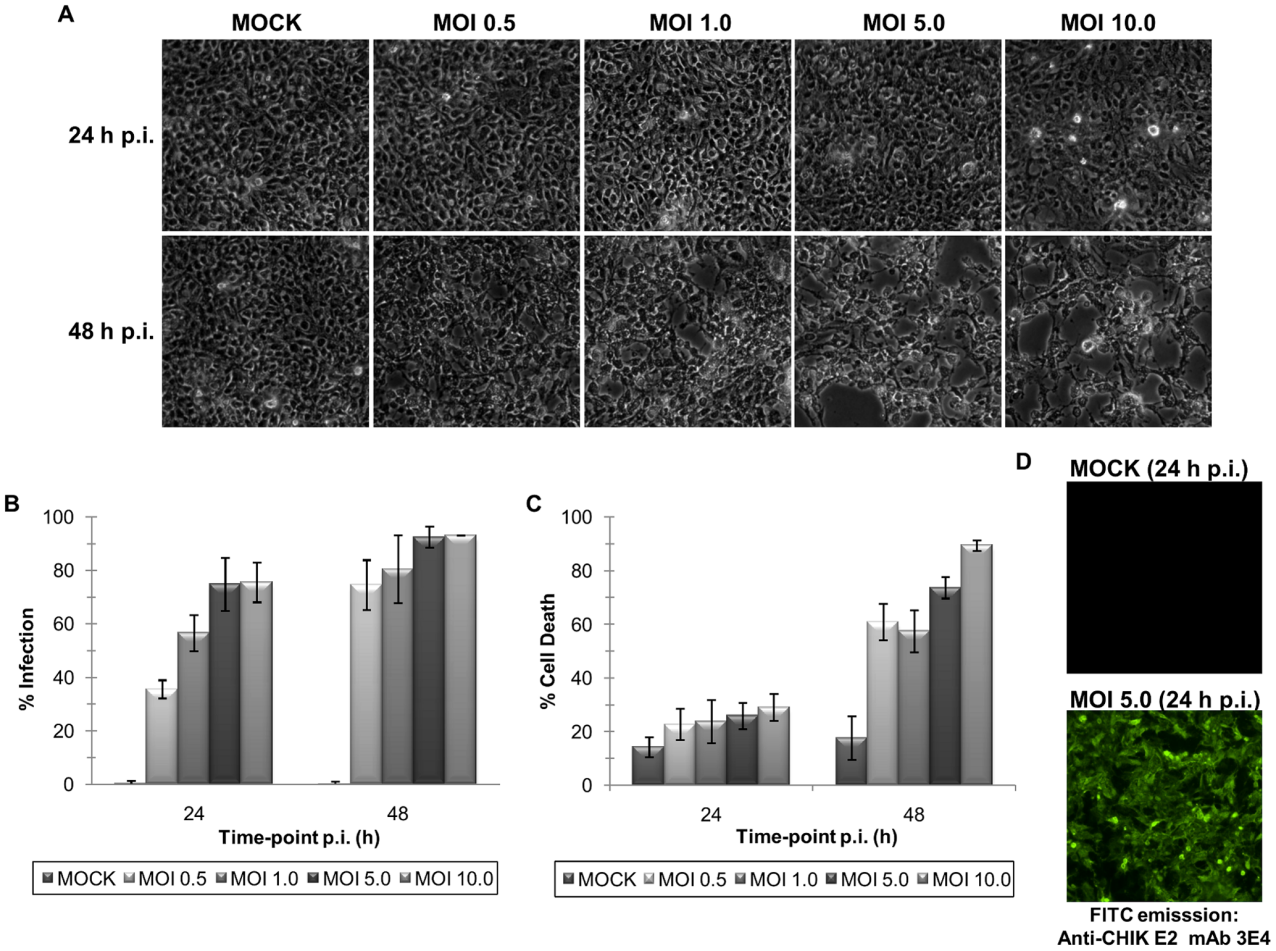
Optimization of the MOI and incubation time-point for early CHIKV infection study. (A) Morphological examination of WRL-68 cells infected at the MOI of 0.5, 1.0, 5.0 and 10.0 at 24 and 48 h incubation revealed a MOI and time-dependent induction of CPE by CHIKV. All images were captured at 100X magnification. (B) Flow cytometric quantification of percentage of cell death by AV/PI double staining of cells. Error bars indicate standard deviation of three biological replicates. (C) Flow cytometric quantification of percentage of infection by immunostaining of cells with anti-CHIK E2 mAB 3E4 (1∶100 dilution). Error bars indicate standard deviation of three biological replicates. (D) Confirmation of infection via indirect immunofluorescence assay at the optimized MOI of 5.0 at 24 h p.i. Mock cells served as negative control. All images were captured at100X magnification.

The cytopathic nature of CHIKV infection in mammalian cell lines, which was reported in several studies [9], [15], [16], was observed in WRL-68 cells infected with the virus at varying MOI (MOI of 0.5, 1.0, 5.0 and 10.0) and time-points (24 and 48 h). This isolate was found to induce cytopathic effects (CPE), characterized by cell shrinkage and detachment, within 48 h of infection, as depicted in Figure 1A. CPE induction was also determined to be MOI-dependent, as cells infected at higher MOI (MOI of 5.0 and 10.0) showed more profound CPE than that of cells infected at low MOI (MOI of 0.5 and 1.0), at 48 h post-infection (p.i.). On the contrary, no significant changes in morphology were observed at 24 h p.i. at the MOI of 0.5, 1.0 and 5.0, while mild CPE was observed at the MOI of 10.0. Mock control cells were cultured in parallel and served as negative control.

### Optimization of the infection conditions for early infection study

As the aim of this study was to investigate alterations in the host cellular proteome during early CHIKV infection (i.e., the stages preceding cell death), the infection conditions (MOI and incubation time-point) were meticulously optimized to maximize infection while maintaining cell death at a minimum level. Relative quantification of percentages of infection and cell death of WRL-68 cells infected at various MOI (MOI of 0.5, 1.0, 5.0 and 10.0) for 24 and 48 h was determined by flow cytometric analysis.

The results showed that WRL-68 cells infected at the MOI of 5.0 for 24 h recorded significantly high percentage of infection at 74.77% (Figure 1B). Percentage of cell death (25.90%), albeit higher than mock control cells (14.33%), showed no significant differences when compared with cells infected at lower MOI (MOI of 0.1 and 0.5) at 24 h p.i. (Figure 1C). Furthermore, prolonging the incubation period significantly increased the percentage of cell death to more than 50%, irrespective of the MOI used. Immunostaining with anti-CHIK E2 mAb 3E4 revealed intense cytoplasmic staining in infected cells at the selected conditions, confirming infection, whereas no staining was apparent with the mock control cells (Figure 1D). Taken together, the MOI of 5.0 and 24 h incubation time-point were determined to be the optimal conditions for early CHIKV infection study.

### 2-DGE profiles of CHIKV infected WRL-68 cells

**Figure 2:**
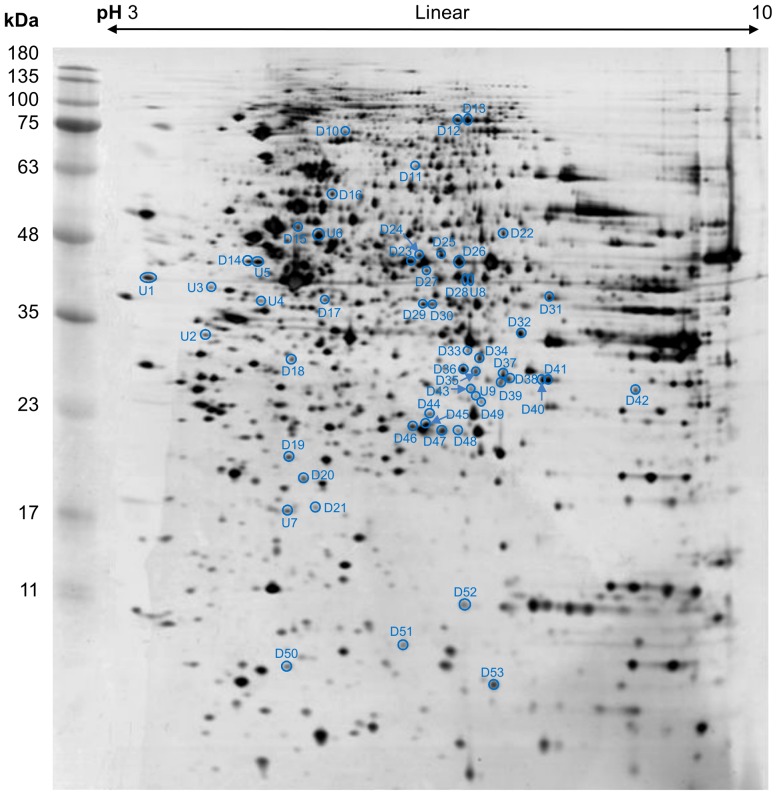
Reference map of the whole cell proteome of WRL-68 cells. Forty µg of protein sample were focused on 13 cm, pH 3–10 linear IPG drystrips, followed by second dimension SDS-PAGE separation on 12.5% polyacrylamide gel which was silver stained. Five biological replicates (n = 5) for each group (Mock control and CHIKV-infected) were analyzed using ImageMaster™ 2D Platinum v7.0 software. Fifty-three spots were determined to be differentially expressed (Fold-change >1.3, *p*<0.05). The position of each spot is indicated by circles on the proteome map. The uppercase ‘U’ and ‘D’ denote up-regulated and down-regulated spots, respectively.

Comparative proteomics analysis between mock control and CHIKV-infected WRL-68 whole cell proteome was carried out using 2-DGE. Five biological replicates (n = 5) were analysed for each group. A typical gel profile for WRL-68 whole cell proteome is shown in Figure 2 (The representative proteome maps for mock control and CHIKV-infected WRL-68 cells are shown in Supplementary Figure S1). Image analysis using the ImageMaster™ 2D Platinum v7.0 software detected more than 1300 spots in each gel. Comparison of the normalized percentage spot volume between both groups revealed 53 differentially expressed spots (Fold-change>1.3, *p*<0.05). Of these, 44 demonstrated reduced spot intensity whereas nine exhibited increased spot intensity. All 53 protein spots were manually excised for subsequent tryptic digestion and tandem MS identification.

### Mass spectrometric identification of differentially expressed proteins

**Figure 3:**
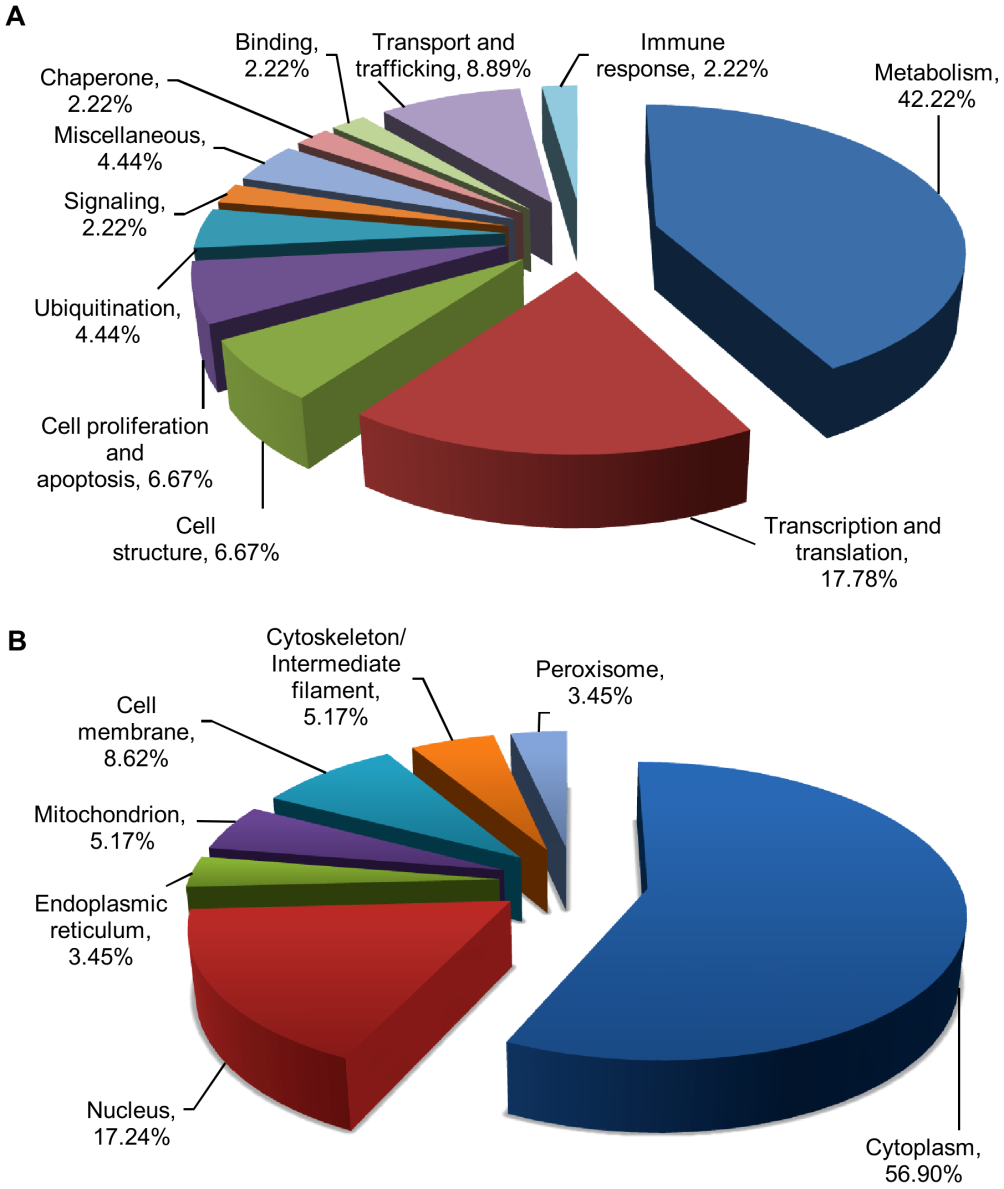
Functional classification and sub-cellular distribution of differentially expressed whole cell proteins during early CHIKV infection. (A) Functional categorization and (B) Sub-cellular localization of differentially modulated proteins were determined based on Swiss-Prot/TrEMBL database search.

Of the 53 protein spots subjected to MALDI-TOF/TOF identification, 50 were successfully identified, corresponding to 45 proteins (Table 1). Unique peptides identified for each protein are listed in Supplementary Table S1. Three protein spots were not identified most likely due to low abundance, resulting in low confidence score. More than one spot was identified for four proteins; guanine nucleotide-binding protein subunit beta-2-like 1 (GNB2L1), Rab GDP dissociation inhibitor beta (GDI2), eukaryotic elongation factor-2 (EEF2) and triosephosphate isomerase (TPI1)). These spots are most likely different isoforms of the protein. Functional classification based on existing information from Swiss-Prot/TrEMBL database identified proteins involved in metabolism (42.22%) and transcription/translation (17.78%) to be mainly affected by CHIKV infection (Figure 3A), whereas classification based on sub-cellular localization showed that most altered proteins to be of cytoplasmic (56.90%) and nuclear origin (17.24%) (Figure 3B).

**Table 1:** List of differentially altered proteins in WLR-68 cells in response to CHIKV infection.

Spot no.	Protein name	Swiss-Prot accession number	MOWSE score/Sequence Coverage (%)	pI/MW^a^ (kDa)	Mock control (Mean ± SD)^b^	CHIKV-infected (Mean ± SD)^b^	Fold-change^c^ (*p*-value)	Peptides matched
U1	Protein SET	Q01105	303 (38)	4.23/33,469	0.0953±0.0250	0.1363±0.0174	1.43 (0.0165)	9
U2	Nucleophosmin (B23)	P06748	100 (22)	4.64/32,555	0.0307±0.0061	0.0426±0.0053	1.38 (0.0113)	4
U3	Reticulocalbin-1	Q15293	299 (30)	4.86/38,866	0.0226±0.0020	0.0319±0.0047	1.41 (0.0038)	9
U4	Heterogeneous nuclear ribonucleoproteins C1/C2	P07910	278 (27)	4.95/33,650	0.0292±0.0186	0.0905±0.0225	3.10 (0.0028)	9
U5	Keratin, type I cytoskeletal 17	Q04695	760 (47)	4.97/48,076	0.1510±0.0178	0.2054±0.0247	1.36 (0.0040)	20
U6	Keratin, type II cytoskeletal 7	P08729	751 (52)	5.50/51,386	0.2186±0.0191	0.3065±0.0551	1.40 (0.0098)	22
U7	Chromobox protein homolog 3	Q13185	88 (27)	5.23/20,798	0.0360±0.0070	0.0501±0.0087	1.39 (0.0222)	4
U8	Pyruvate dehydrogenase E1 component subunit alpha, mitochondrial	P08559	126 (19)	8.35/43,268	0.0395±0.0198	0.0773±0.0234	1.96 (0.0249)	7
D10	Spartin	Q8N0X7	80 (7)	5.66/72,788	0.0256±0.0073	0.0154±0.0053	–1.67 (0.0346)	4
D11	Phosphoglucomutase-2	Q96G03	197 (26)	6.28/68,240	0.0269±0.0079	0.0118±0.0035	–2.29 (0.0044)	13
D12	Elongation factor-2	P13639	497 (37)	6.42/95,277	0.1986±0.0170	0.1470±0.0450	–1.35 (0.0430)	27
D13	Elongation factor-2	P13639	338 (20)	6.42/95,277	0.1261±0.0239	0.0774±0.0303	–1.63 (0.0224)	11
D14	Gamma-enolase	P09104	215 (36)	4.91/47,239	0.0873±0.0115	0.0592±0.0105	–1.47 (0.0037)	13
D15	Hydroxymethylglutaryl-CoA synthase, cytoplasmic	Q01581	62 (17)	5.22/57,257	0.0655±0.0143	0.0405±0.0037	–1.62 (0.0053)	8
D16	Copine-1	Q99829	147 (4)	5.52/59,022	0.0436±0.0154	0.0249±0.0038	–1.75 (0.0299)	3
D18	Spermidine synthase	P19623	228 (23)	5.30/33,803	0.0390±0.0067	0.0290±0.0059	–1.35 (0.0360)	6
D19	Ubiquitin-conjugating enzyme E2 N	P61088	215 (39)	5.33/22,393	0.0420±00028	0.0290±0.0038	–1.45 (0.0003)	6
D20	Inosine triphosphate pyrophosphatase	Q9BY32	97 (27)	5.50/21,432	0.0391±0.0034	0.0296±0.0057	–1.32 (0.0121)	3
D21	Adenine phosphoribosyltransferase	P07741	239 (57)	5.78/19,595	0.0214±0.0054	0.0141±0.0036	–1.52 (0.0355)	7
D22	Nicotinamide phosphoribosyltransferase	P43490	164 (15)	6.69/55,487	0.0527±0.0085	0.0365±0.0069	–1.44 (0.0106)	8
D23	Rab GDP dissociation inhibitor beta	P50395	441 (42)	6.11/50,631	0.0894±0.0156	0.0382±0.0226	–2.34 (0.0032)	14
D24	Rab GDP dissociation inhibitor beta	P50395	132 (34)	6.11/50,631	0.0575±0.0136	0.0381±0.0105	–1.51 (0.0363)	11
D25	La ribonucleoprotein	P05455	101 (23)	6.68/46,808	0.0886±0.0226	0.0128±0.0531	–1.67 (0.0159)	10
D26	Alpha-enolase	P06733	113 (28)	7.01/47,139	0.2530±0.0202	0.1909±0.0269	–1.32 (0.0033)	9
D27	Adenylosuccinate synthetase isozyme 2	P30520	486 (46)	6.13/50,066	0.0503±0.0163	0.0223±0.0120	–2.25 (0.0148)	16
D28	Isocitrate dehydrogenase, cytoplasmic	O75874	176 (24)	6.53/46,630	0.1428±0.0320	0.0910±0.0199	–1.57 (0.0152)	10
D29	Eukaryotic translation initiation factor 3 subunit H	O15372	96 (11)	6.09/39,905	0.0702±0.0155	0.0444±0.0152	–1.58 (0.0286)	4
D30	Poly(rC)-binding protein1 (hnRNP E1)	Q15365	116 (29)	6.66/37,474	0.0698±0.0094	0.0492±0.0163	–1.42 (0.0393)	7
D31	Phosphoserine aminotransferase	Q9Y617	81 (14)	7.56/40,397	0.0956±0.0262	0.0553±0.0150	–1.73 (0.0176)	5
D32	Aldo-keto reductase family 1 member C2	P52895	250 (40)	7.13/36,712	0.0873±0.0164	0.0541±0.0176	–1.62 (0.0148)	9
D33	Pirin	O00625	107 (33)	6.42/32,093	0.0198±0.0028	0.0126±0.0119	–1.57 (0.0052)	8
D34	Ribose-phosphate pyrophosphokinase1	P60891	104 (38)	6.51/34,812	0.0668±0.0072	0.0504±0.0027	–1.33 (0.0014)	9
D35	Glucosamine-6-phosphate isomerase 1	P46926	95 (27)	6.42/32,648	0.0652±0.0141	0.0455±0.0082	–1.43 (0.0273)	7
D36	S-formylglutathione hydrolase	P10768	68 (31)	6.54/31,442	0.1085±0.0110	0.0737±0.0158	–1.47 (0.0037)	7
D37	Actin-related protein 2/3 complex subunit 2 (p34-ARC)	O15144	192 (42)	6.84/34,311	0.1003±0.0138	0.0710±0.0118	–1.41 (0.0069)	12
D38	Electron transfer flavoprotein subunit alpha, mitochondrial	P13804	129 (22)	8.62/35,058	0.0639±0.0130	0.0450±0.0124	–1.42 (0.0467)	5
D39	Guanine nucleotide-binding protein subunit beta-2-like 1	P63244	64 (18)	7.60/35,055	0.0417±0.0078	0.0162±0.0032	–2.58 (0.0016)	5
D40	Guanine nucleotide-binding protein subunit beta-2-like 1	P63244	400 (58)	7.60/35,055	0.1428±0.0483	0.0758±0.0361	–1.88 (0.0379)	15
D41	Guanine nucleotide-binding protein subunit beta-2-like 1	P63244	606 (67)	7.60/35,055	0.1226±0.0117	0.0871±0.0182	–1.40 (0.0064)	16
D42	Cyclin-dependent kinase 1	P06493	335 (58)	8.37/34,047	0.0426±0.0112	0.0240±0.0054	–1.77 (0.0102)	13
D43	Translation initiation factor eIF-2B subunit alpha	Q14232	94 (34)	6.90/33,691	0.0375±0.0071	0.0243±0.0047	–1.54 (0.0084)	8
D44	Phosphoglycerate mutase 1	P18669	197 (26)	6.28/68,240	0.0450±0.0043	0.0327±0.0084	–1.37 (0.0197)	8
D45	Proteasome subunit alpha type-6	P60900	215 (50)	6.34/27,382	0.0964±0.0120	0.0635±0.0173	–1.52 (0.0081)	10
D46	Isopentyl-diphosphate Delta-isomerase 1	Q13907	129 (14)	5.93/26,302	0.0643±0.0075	0.0395±0.0077	–1.63 (0.0008)	2
D47	Triosephosphate isomerase	P60174	141 (55)	6.45/26,653	0.1147±0.0132	0.0778±0.0089	–1.81 (0.0008)	10
D48	Triosephosphate isomerase	P60174	129 (33)	6.45/26,653	0.0416±0.0106	0.0230±0.0032	–1.47 (0.0055)	5
D49	S-methyl-5-thioadenosine phosphorylase	Q13126	303 (53)	6.75/31,230	0.0261±0.0049	0.0182±0.0053	–1.44 (0.0396)	11
D50	Thioredoxin-like protein 5	Q9BRA2	103 (22)	5.40/13,932	0.0366±0.0036	0.0268±0.0067	–1.37 (0.0203)	2
D51	Fatty-acid binding protein, epidermal	Q01469	100 (52)	6.60/15,155	0.0389±0.0081	0.0268±0.0021	–1.46 (0.0117)	7
D52	Peptidyl-prolyl cis-trans isomerase A (Cyclophilin A)	P62937	158 (41)	7.68/18,001	0.0543±0.0035	0.0399±0.0066	–1.36 (0.0025)	8

a MW and pI refer to the molecular weight and isoelectric point of the protein.

b The mean % spot volume (n = 5) was used for the analysis of fold difference between mock control and CHIKV-infected protein spots. SD represents standard deviation of five biological replicates.

c Positive fold-change values represent up-regulation whereas negative fold-change values signify down-regulation of identified proteins.

### Protein network analysis

**Figure 4:**
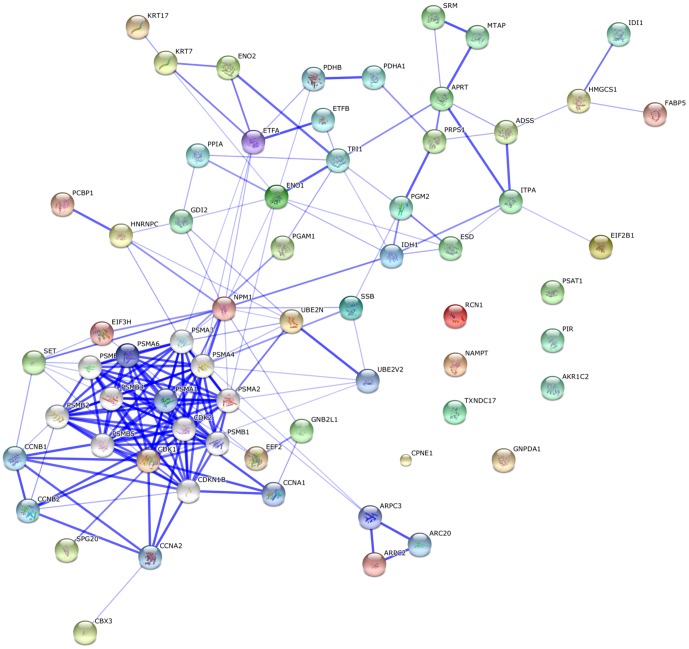
STRING interaction network showing association between differentially expressed proteins. Interaction map was generated using default settings (Medium confidence of 0.4 and 7 criteria for linkage: neighbourhood, gene fusion, co-occurrence, co-expression, experimental evidences, existing databases and text mining). Twenty additional interplay proteins were also added to each network. The protein names and gene symbols used in this network are listed in Supplementary Table S2.

STRING network analysis of protein-protein interactions was performed to identify functionally linked proteins and determine the potential biological processes affected [17]. The network is presented under confidence view, whereby stronger associations are represented by thicker lines or edges and vice versa, whereas proteins are represented as nodes. Twenty additional interacting proteins were added to provide a more comprehensive view of the interactions. The protein names and gene symbols used in this network are listed in Supplementary Table S2. All gene symbols were derived from the HUGO Gene Nomenclature Committee (HGNC) (http://www.genenames.org). Figure 4 shows the interaction between 45 identified proteins and the additional interactors. Thirty seven proteins were found to be linked either directly or indirectly through one or more interacting proteins, suggesting the existence of reported functional linkages. Eight biological processes were determined to be significantly involved (*p*<0.05 based on false discovery rate (FDR) correction) in this network, including energy production, cell cycle regulation, gene expression, mRNA metabolism, protein metabolism and modification, DNA replication and ubiquitin-protein ligase activity (Table 2).

**Table 2:** GO enrichment analysis of the biological processes involved in the STRING protein network.

GO Biological process	*p*-value^a^
Regulation of ubiquitin-protein ligase activity	4.34×10^−14^
Gene expression	1.14×10^−6^
mRNA metabolic process	8.06×10^−6^
Protein modification	1.75×10^−5^
Regulation of cell cycle	1.63×10^−5^
Protein metabolic process	1.02×10^−5^
Generation of energy and precursor metabolite	1.35×10^−2^
DNA replication	5.05×10^−2^

a The significance of the GO biological process derived from the cytosolic protein network was determined by FDR correction (*p*<0.05).

### Immunoblot validation of proteomics data

**Figure 5:**
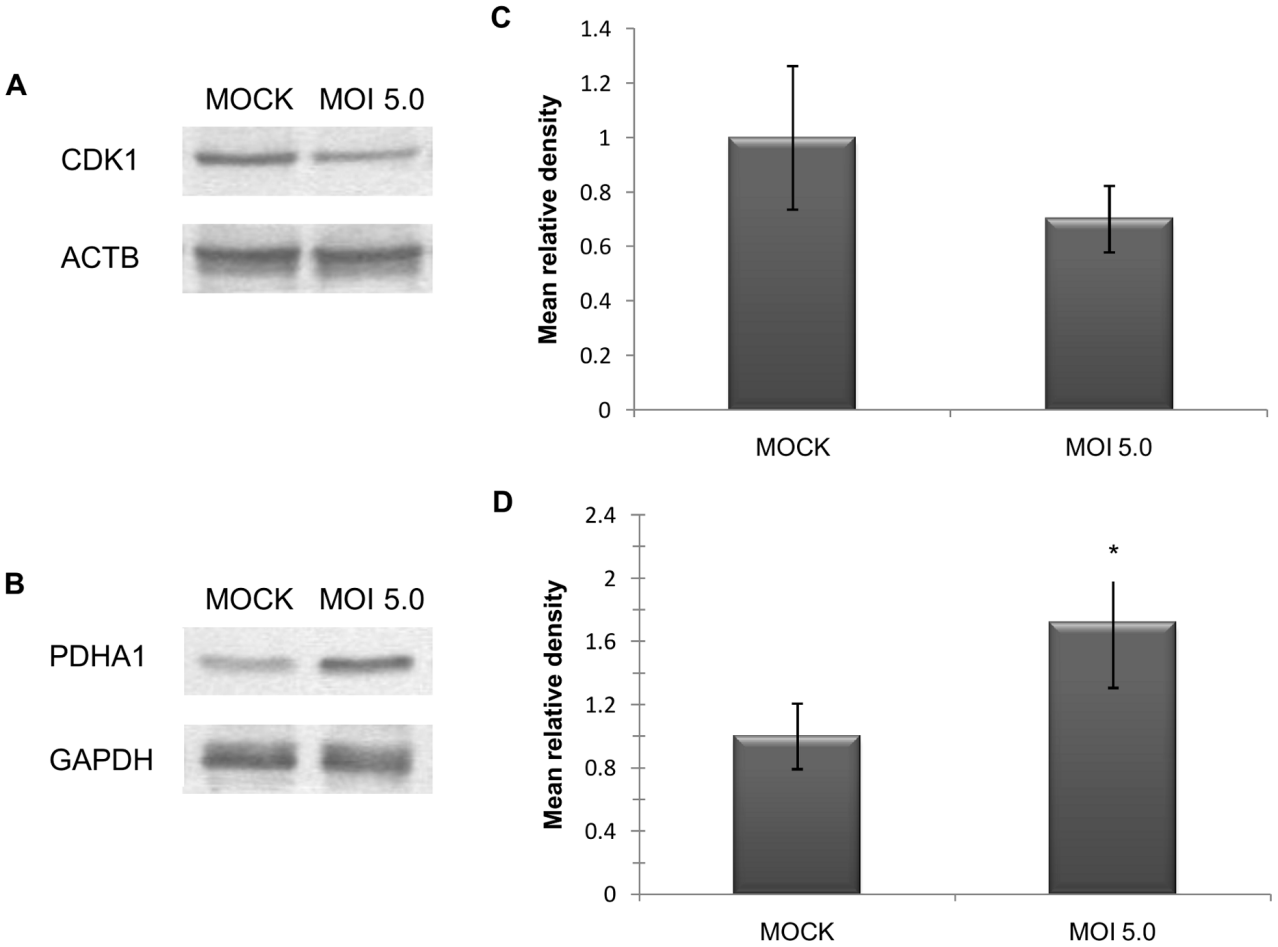
Western blot validation and densitometric analysis of CDK1 and PDHA1 proteins. Confirmation of the expression profiles for CDK1 (A) and PDHA1 (B) was performed via immunoblot analysis. Densitometric analysis of the mean relative intensity (n = 3) for each target protein showed down-regulation of CDK1 by 1.42 fold (C) and up-regulation of PDHA1 by 1.72 fold (D). The intensity for CDK1 and PDHA1 was normalized against ACTB and GAPDH, respectively. Error bars indicate standard deviation of three biological replicates. * indicates significant difference in expression (*p*<0.05).

Two proteins, CDK1 and PDHA1, representing the down- and up-regulated groups respectively, were randomly selected for Western blot validation. GAPDH was used as the loading control for PDHA1 as both PDHA1 and ACTB have similar molecular mass of ∼43 kDa, and thus, cannot be stained together on the same blot. Immunoblots confirmed their down- and up-regulation, as shown in Figures 5A and 5B. Densitometric analysis revealed fold differences of −1.42 and 1.72 CDK1 and PDHA1 respectively (Figures 5C and 5D), which was comparable to the observed −1.77 and 1.96 fold-changes in 2-DGE analysis.

### Transcript expression analysis of selected altered proteins

The transcript expression of 36 selected proteins was evaluated using real-time qPCR (The gene names and primer sequences are listed in Supplementary Table S3). All primers had amplification efficiencies within the acceptable range of 90 to 110% (Slope values between −3.1 to −3.6). It is a known fact that mRNA expression do not always correlate with protein expression [18]. In our study however, the direction of mRNA and protein expression changes of 15 proteins including CDK1 and PDHA1 were the same (Table 3). On the other hand, the transcript expression of four other proteins; adenine phosphoribosyltransferase (APRT), electron transport flavoprotein subunit alpha (ETFA), actin-related protein 2/3 complex subunit 2 (ARPC2) and cyclophilin A (PPIA), showed the opposite direction of expression change despite being statistically significant. Meanwhile, the mRNA expression levels of 17 other proteins showed no statistically significant differences.

**Table 3:** Comparison of real-time qPCR and proteomics results for selected genes.

Gene symbol	mRNA fold-change	Protein fold-change
**UBE2N**	**−1.33**	**−1.45**
PSMA6	NSD	−1.52
**SET**	**1.41**	**1.43**
GNB2L1	NSD	**−**2.58, −1.88, −1.40**
**CDK1**	**−1.38**	**−1.77**
**PDHA1**	**1.30**	**1.96**
**ENO1**	**−1.32**	**−1.32**
**IDH1**	**−1.14**	**−1.57**
PGAM1	NSD	−1.37
TPI1	NSD	−1.81, −1.47**
HMGCS1	NSD	−1.62
**IDI1**	**−1.48**	**−1.63**
NAMPT	NSD	−1.44
ITPA	NSD	−1.32
APRT	1.33	−1.52
**ADSS**	**−1.36**	**−2.25**
**PRPS1**	**−1.27**	**−1.33**
**MTAP**	**−1.26**	**−1.44**
**PSAT1**	**−1.62**	**−1.73**
CBX3	NSD	1.39
**PIR**	**−1.25**	**−1.57**
**EEF2**	**−1.12**	**−1.63, −1.35** **
EIF3H	NSD	−1.58
**EIF2B1**	**−1.25**	**−1.54**
HNRNPC	NSD	3.1
**PCBP1**	**−1.82**	**−1.42**
SSB	NSD	−1.67
KRT7	NSD	1.4
ARPC2	1.24	−1.41
NPM1	NSD	1.38
CPNE1	NSD	−1.75
GDI2	NSD	−2.34, −1.51**
ETFA	1.34	−1.42
PPIA	1.14	−1.36
RCN1	NSD	1.41
TXNDC17	NSD	−1.37

* **Bold** indicates RNA expression changes which are in concordance with protein expression changes in terms of directionality, and are determined to be statistically significant (*p*<0.05); NSD indicates no significant differences in the RNA expression.

** More than one protein spot was identified.

## Discussion

It is well-established that CHIKV induces rapid and profound CPE in human host cells which culminate in cell death via apoptosis. The events preceding the inevitable cell demise, however, remain ill-characterized. A previous proteomic study on new-born mice focused on investigating the dynamic overview of altered protein expression during late stages of CHIKV infection, whereby alterations of stress, inflammation, urea cycle, energy metabolism and apoptotic-related proteins were implicated in the observed disease pathogenesis [13]. In this study, we shifted the focus to examining global changes of the host cell proteome during early CHIKV infection, with aims of identifying key proteins that are potentially involved in facilitating CHIKV replication. It has been reported that during early infection, viral replication and dissemination occurs rapidly through manipulation of the host cell machinery owing to the simplicity of the viral makeup [19]. By collating data from proteomics and bioinformatics analyses, we inferred the potential manipulation or subversion of various important cellular processes including mRNA and protein metabolism, energy production, ubiquitin-proteasome pathway (UPP) and cell cycle regulation by CHIKV.

### Alteration of proteins involved in mRNA processing and translation machinery

Virus hijacking of the host mRNA processing and translational machinery is an essential process for virus replication. Viruses with positive sense RNA in particular, have been shown to recruit components of the host protein biosynthesis machineries for viral RNA and protein synthesis [20]. In the current study, we identified several deregulated proteins involved in mRNA processing and translation, including heterogeneous nuclear ribonucleoproteins C1/C2 (hnRNP C1/C2), poly(rC)-binding protein 1 (hnRNP E1), elongation factor- 2 (EEF-2), translation initiation factor EIF-2B subunit alpha (eIF2B1) and eukaryotic translation initiation factor 3 subunit H (eIF3H).

Heterogeneous ribonucleoproteins (hnRNPs) are complexes of RNA and proteins involved in an array of cellular functions such as transcription, pre-mRNA processing and cytoplasmic mRNA translation and turnover [21]. In our study, hnRNP C1/C2 was found to be up-regulated by 3.10 fold while hnRNP E1 was down-regulated by 1.42 fold. Transcript level of hnRNP E1 mRNA showed similar down-regulation while the mRNA expression of hnRNP C1/C2 was not significantly altered, suggesting that post-transcriptional and post-translational modification may play a role in modulating the expression of the latter protein. In a previous study, hnRNP C1/C2 was shown to promote dengue virus survival in host cells [22] while hnRNP E1 inhibits vesicular somatitis virus replication [23]. Ergo, the up-regulation of hnRNP C1/C2 in the present study may signify its recruitment by CHIKV whereas hnRNP E1 may possibly exert negative effects towards CHIKV propagation which is counteracted by its inhibition.

Translation factors are known to play crucial roles in viral RNA and protein synthesis and different viruses exert different mechanisms to modulate host translational proteins to their benefit, as shown in several studies [24], [25], [26]. Alphaviruses have been shown to induce global shutoff of protein synthesis by inhibiting or modifying host translational factors [27]. CHIKV-induced host translational shutoff was recently shown to occur, through an unidentified protein kinase R (PKR)-independent mechanism [28]. In this study, down-regulation of proteins involved in initiation of translation (eIF2B1 and eIF3SH) and elongation of the newly synthesised polypeptide chain (EEF-2) was observed, although at the transcript level, only EEF-2 and eIF2B1 genes were down-regulated. The exact roles of these proteins in host translational shutoff, however, cannot be ascertained at this point. Nonetheless, down-regulation of these proteins may inhibit the host translational machinery to a certain extent, possibly contributing to the observed down-regulation of most altered proteins in this study.

### Differential expression of proteins involved in cellular energy production and metabolism

Of the 19 regulated proteins identified to be involved in cellular metabolism, 18 were down-regulated. Only PDHA1, a subunit of the pyruvate dehydrogenase complex involved in transforming pyruvate to acetyl-CoA in the tricarboxylic acid (TCA) cycle [29], was up-regulated by 1.96 fold. Up-regulation of this protein was further confirmed by immunoblot (Figures 5B and 5D). Transcript expression study on 14 selected genes revealed that 8 genes; PHDA1, alpha-enolase (ENO1), isocitrate dehydrogenase (IDH1), isopentyl-diphosphate Delta-isomerase 1 (IDI1), adenylosuccinate synthetase isozyme 2 (ADSS), ribose-phosphate pyrophosphokinase 1 (PRPS1), S-methyl-5-thioadenosine phosphorylase (MTAP) and phosphoserine aminotransferase (PSAT1), had expression changes of the same directionality as the protein expression (Table 1).

Based on the proteomics analysis, energy production in WRL-68 cells was expected to be significantly affected through reduced expression of glycolytic enzymes including ENO1, TPI1 and phosphoglycerate mutase 1 (PGAM1), as well as down-regulation of IDH1 which catalyzes the oxidative decarboxylation of isocitrate to alpha-ketoglutarate in the TCA cycle [30]. Four proteins associated with the adenine salvage pathway, namely PRPS1, ADSS, MTAP and adenine phosphoribosyltransferase (APRT), were also down-regulated. Similar dysregulation was observed with IDI1 and hydroxymethylglutaryl-CoA synthase (HMGCS1), two key enzymes involved in the biosynthesis of cholesterol, coenzyme Q and isoprenylated proteins through the mevalonate pathway [31].

### Effects on proteins involved in the UPP

UPP is an essential intracellular system for protein degradation, with multiple cellular functions including cell cycle regulation, apoptosis, DNA repair, signal transduction and transcriptional regulation [32]. Many viruses have been reported to evolve different strategies to utilize the UPP for various purposes, including avoidance of host immune surveillance, viral maturation, viral progeny release and transcriptional regulation [33], [34], [35]. Our proteomics data showed down-regulation of two UPP associated proteins; ubiquitin-conjugating enzyme E2 N (UBE2N) and proteasome subunit alpha type-6 (PSMA6). At the transcript level however, only UBE2N showed the same direction of expression change as the protein expression. UBE2N is a ubiquitin-carrier enzyme which carries and binds ubiquitin to the ubiquitin-ligase enzyme for subsequent ubiquitination of targeted proteins. PSMA6 is the subunit of the 20S proteasome subcomplex which forms the multicatalytic 26S proteasome that degrades polyubiquitinated proteins into smaller peptides [32].

### Down-regulation of proteins involved in cell cycle regulation

Cyclin-dependent kinases (CDKs) are a family of cyclin-activated serine/threonine kinases involved in various cellular processes including regulation of cell cycle (CDK1, 2, 3, 4, 6 and 7), neuronal functions (CDK5) and transcription (CDK7, 8 and 9) [36]. While CDKs are commonly associated with nuclear replication of DNA and RNA viruses, several studies have expanded the role of CDKs to cytoplasmic replication of RNA viruses as well [37], [38]. In this study, CDK1 was found to be down-regulated, both at the protein and gene expression level. CDK1 is activated by cyclin B and functions in allowing entry into mitosis from the G2 phase [39]. Inhibition of this protein would cause cell cycle arrest at G2 phase. Meanwhile, SET protein is a phosphoprotein found to regulate the cell cycle by inhibiting cyclin B-CDK1 activity [40]. In our study, SET protein was found to be up-regulated at both the protein and transcript levels, which favours the inhibition of cyclin B-CDK1 activity.

In conclusion, our proteomics data suggested that during early infection, CHIKV affects the expression of proteins involved in mRNA processing, host metabolic machinery, UPP, and cyclin-dependent kinase 1 (CDK1) regulation (in favour of virus survival, replication and transmission). While results from this study complement the proteomics results obtained from previous late host response studies, functional characterization of these proteins is warranted to reinforce our understanding of their roles during early CHIKV infection in humans.

## Materials and Methods

### Cell lines

WRL-68 human hepatic cells, a HeLa derivative cell line that is highly susceptible to CHIKV infection (ATCC Cat No. CL-48), Vero cells (ATCC Cat. No. CCL-81), and C6/36 *Aedes albopictus* cells (ATCC Cat. No. CRL-1660) were used in this study. WRL-68 and Vero cells were cultured in DMEM medium (GIBCO, Grand Island, NY) supplemented with 10% heat-inactivated fetal bovine serum (FBS) (GIBCO, Grand Island, NY) at 37 °C. C6/36 cells were grown in L-15 medium (Sigma Aldrich, St Louis, MO) supplemented with 10% tryptose phosphate broth (TPB) (Sigma Aldrich, St Louis, MO) and 10% FBS at 28 °C.

### Antibodies

The antibodies used for indirect immunofluorescence assay (IIFA) and immunostaining by flow cytometry were anti-CHIK E2 monoclonal antibody (mAb) 3E4 (a kind gift from Dr. Philippe Desprès from the Pasteur Institute of France) and FITC-conjugated goat anti-mouse IgG secondary antibody (Novus Biologicals, Littleton, CO). The primary antibodies used for Western blot validation were mouse mAb to beta-actin (ACTB), cyclin-dependent kinase 1 (CDK1), glyceraldehyde 3-phosphate dehydrogenase (GAPDH) or pyruvate dehydrogenase (PDHA1). Horseradish peroxidise (HRP)-conjugated goat anti-mouse IgG was used as the secondary antibody. All antibodies used for validation were purchased from Santa Cruz Biotechnology, Santa Cruz, CA.

### Virus stock propagation and titration

CHIK/06/08 clinical isolate of the ECSA genotype was propagated twice in C6/36 cell line and virus stock was harvested from the culture supernatant and stored at −80 °C. Mock control cells were cultured in parallel but without virus infection and processed in the same manner. Virus titer was determined by standard plaque assay procedure on Vero cells. Titers were expressed as plaque-forming units (PFU)/ml.

### Infection of WRL-68 cells with CHIKV

WRL-68 cells were infected with CHIKV at the MOI of 0.5, 1.0, 5.0 and 10.0 for 2 h at 37 °C. Mock control cells were incubated in parallel with culture supernatant of mock control C6/36 cells. Viral inoculum was subsequently removed and the cells were further incubated in DMEM maintenance medium containing 2% FBS for 24 and 48 h. The optimal MOI and time-point for early infection study were selected based on flow cytometric quantitative analysis of percentage of cell infection and cell death [24].

### IIFA

Prior to flow cytometric quantification, CHIKV infection in WRL-68 cells was confirmed by IIFA, as previously described [41] with modifications. WRL-68 cells were seeded overnight at a density of 1.5×10^5^ cells/well in a 24-well culture dish, and subsequently infected at various MOI. Mock control cells were cultured in parallel. After 24 and 48 h incubation, the cells were fixed with 3.7% formaldehyde in phosphate buffered saline (PBS) for 20 min, washed with PBS and permeabilized with 0.15 M glycine for 10 min. Permeabilized cells were washed extensively and further incubated with anti-CHIK E2 mAb 3E4 (1∶100 dilution) for 30 min at 37 °C. Thereafter, the cells were washed with PBS and incubated in FITC-conjugated secondary antibody (1∶1000 dilution) for 30 min at 37 °C. The cells were observed under an inverted microscope (Nikon Eclipse Ti-5, Japan) and fluorescent pictures were acquired using NIS-Elements imaging software (Nikon, Japan).

### Flow cytometric quantification of percentage CHIKV infection and cell death

Quantification of percentage infection was carried out as previously described [24] with modifications. Mock control and CHIKV-infected cells were harvested at appropriate time-points and fixed with 3.7% formaldehyde for 30 min. The cells were washed with staining buffer (0.1% (w/v) sodium azide in 1% FBS, pH 7.5), and incubated with anti-CHIK E2 mAb 3E4 (1∶100 dilution) for 90 min at 37 °C. Thereafter, the cells were washed and further incubated in FITC-conjugated goat anti-mouse IgG secondary antibody (1∶1000 dilution) for 60 min at 37 °C. After extensive washing, the cells were resuspended in PBS and analyzed with BD FACSCanto II flow cytometer (BD Biosciences, San Jose, CA) using FACSDiva v6.1 software.

Percentage cell death was determined using FITC Annexin V Apoptosis Detection Kit I (BD Biosciences, San Jose, CA) according to the manufacturer's protocol. Annexin V/propidium iodide stained cells were analyzed by flow cytometry.

### Protein sample processing

Whole cell proteome were extracted on ice with lysis buffer (7 M Urea, 2 M Thiourea, 4% CHAPS, 2% IPG Buffer, 40 mM DTT). Cellular debris was pelleted at 17,000×*g* and protein supernatant was cleaned using 2-D Clean-Up Kit (GE Heathcare, Uppsala, Sweden) as described by the manufacturer. Protein estimation was performed using Bradford Protein assay (Bio-Rad Laboratories, Richmond, CA).

### 2-DGE

Forty µg (for analytical gel) and 160 µg (for preparative gel) of protein was mixed with rehydration buffer (7 M Urea, 2 M Thiourea, 2% CHAPS, 0.5% IPG Buffer, 1% Bromophenol blue) to a final volume of 250 µl and left overnight to rehydrate into 13 cm pH 3–10 linear immobilized pH gradient DryStrips (GE Healthcare, Uppsala, Sweden). First dimension isoelectric focusing was performed at 20 °C according to the following protocol: (i) 500 Vh, 500 V (Step-and-hold), (ii) 1,000 Vh, 1,000 V (Gradient), (iii) 16,000 Vh, 8,000 V (Gradient) and (iv) 12,000 Vh, 8,000 V (Step-and-hold). The strips were subsequently equilibrated with equilibration buffer (6 M Urea, 75 mM Tris-HCl pH 8.8, 29.3% Glycerol, 2% SDS, 0.002% Bromophenol Blue) containing 1% DTT for 15 min, followed by equilibration with equilibration buffer containing 2.5% iodoacetamide for another 15 min. Proteins were resolved on 12.5% SDS-PAGE homogenous gels at 50 V for 30 min, and 500 V for 2 h. Gels were silver stained according to a modified, MS-compatible silver staining protocol [42].

### Differential gel analysis

Gels were scanned with ImageScanner™ III (GE Healthcare, Uppsala, Sweden) and analyzed using ImageMaster™ 2D Platinum v7.0 software (Amersham Biosciences, Sweden). Ten gels were used for analysis (five biological replicates per group). The volume of each spot was normalized against the total volume of all spots in the gel, and the normalized values were expressed as percentage spot volume. Spots having a fold-change of at least 1.3 and *p*<0.05 (as determined by one-way ANOVA and Student's *t*-test) were excised from multiple preparative gels for in-gel digestion.

### In-gel tryptic digestion

In-gel digestion was performed using Trypsin Gold (Promega, Madison, WI) as previously described [43], [44]. Briefly, excised spots were destained with destaining solution (15 mM potassium ferricyanide/50 mM sodium thiosulphate), followed by reduction with 10 mM DTT/100 mM ammonium bicarbonate for 30 min at 60 °C and alkylation with 55 mM iodoacetamide/100 mM ammonium bicarbonate for 20 min in the dark. The gel plugs were washed trice with 50% acetonitrile (ACN)/100 mM ammonium bicarbonate, 20 min each wash, and dehydrated with 100% ACN for 20 min. The gel plugs were subsequently dried using a vacuum centrifuge (HetoVac VR-1 vacuum concentrator, Birkercd, Denmark), and digested overnight in 25 µl of 10 ng/µl trypsin at 37 °C. Tryptic peptides were then extracted twice, first with 50% ACN for 15 min, followed by 100% ACN for another 15 min. The extracted solutions were pooled together into a clean tube and dried using a vacuum centrifuge.

### MALDI-TOF/TOF analysis

Dried peptides were reconstituted in 0.1% formic acid (FA) and desalted using ZipTip C18 (Millipore, Billerica, USA), according to the manufacturer's protocol. Following ZipTip cleanup, the peptides were eluted out in 2 µl elution solution (50% ACN/0.1% FA) and mixed with saturated α-cyano-4-hydroxycinnamic acid (CHCA) matrix prepared in 50% ACN/0.1% trifluoroacetic acid (TFA), at a 1∶1 ratio. Peptides were spotted on stainless-steel sample target plate in 0.7 µl aliquots in duplicates. Mass spectra for each peptide were obtained on a MALDI-TOF/TOF (ABI 4800 Plus, Applied Biosystems™, Foster City, CA) mass spectrometer using a previously established setting [43]. The spectra were analyzed with the Global Protein Server (GPS) explorer 3.6 software (Applied Biosystems™, Foster City, CA), which uses an internal MASCOT program (Matrix Science, London, UK) to match the MS and MS/MS data against existing database information. The data obtained were searched against human databases downloaded from the Swiss-Prot/TrEMBL homepage (http://www.expasy.ch/sprot).

### Bioinformatics

Categorization of functional and sub-cellular distribution of proteins was performed based on Swiss-Prot/TrEMBL database search. Protein-protein interactions were predicted using Search Tool for the Retrieval of Interacting Genes/Proteins (STRING) database v9.0 (http://www.string-db.org/). The Swiss-Prot identifier for the genes (eg. ENOA_HUMAN for alpha-enolase), in ‘Protein mode’, was used to search against the STRING database. Network analysis was set at medium stringency (STRING score = 0.4). Proteins were linked based on seven criteria; neighbourhood, gene fusion, co-occurrence, co-expression, experimental evidences, existing databases and textmining.

### Western blot

Samples of CHIKV-infected and mock control cells from three independent biological replicates (not used for 2-DGE analysis) were lysed with RIPA buffer (25 mM Tris pH 7.6, 150 mM NaCl, 1.0% Triton-X, 1.0% Sodium deoxycholate, 0.1% SDS) and quantified using BCA Protein Assay Kit (Pierce, Rockford, IL). Denatured proteins (20 µg) from each sample were loaded into each lane and resolved on 12.5% polyacrylamide gels at a constant voltage of 100 V. The resolved proteins were electroblotted onto PVDF-membranes at a constant current of 80 mA for 1 h 30 min. Non-specific bindings were blocked overnight at 4 °C with 5% w/v non-fat powdered milk in Tris-buffered saline with Tween-20 (TBST) solution (50 mM Tris pH 7.4, 150 mM NaCl, 0.05% Tween-20). After extensive washing, the membranes were incubated with either mouse mAb to ACTB, CDK1, GAPDH or PDHA1 (1∶500 dilution) for 1 h 30 min at room temperature. Subsequently, the membranes were incubated with HRP-conjugated goat anti-mouse IgG (1∶2,500 dilution) for 1 h at room temperature. Target proteins were detected with TMB Stabilized Substrate for HRP (Promega, Madison, WI). The blots were scanned using ImageScanner™ III in reflective mode and densitometric quantification was performed using ImageJ v1.45 freeware (http://rsbweb.nih.gov/ij). The mean relative density for each target band was normalized against ACTB or GAPDH.

### Real-time quantitative PCR (qPCR)

Total RNA of CHIKV-infected and mock control cells from three biological replicates was extracted using Qiagen RNeasy Mini Kit (Qiagen, Valencia, CA) as described by the manufacturer. Purity of extracted RNA was determined by measuring the A260/A280 and A230/A260 absorbance ratio using GeneQuant™ 1300 spectrophotometer (GE Healthcare, Uppsala, Sweden). RNA integrity was confirmed by visualization of distinct 18S and 28S ribosomal RNA bands resolved on 1% agarose gel electrophoresis. One µg of high quality RNA was converted to cDNA using High Capacity RNA-to-cDNA Kit (Applied Biosystems™, Foster City, CA), following the manufacturer's protocol. Primers specific for the gene of interest were designed with Primer3 Input v4.0 (http://frodo.wi.mit.edu/primer3/) and primer efficiency test was performed for each primer pair to confirm specificity towards the gene of interest. RNA sample (10 ng) was mixed with the respective primer pair and Fast SYBR®Green Master Mix (Applied Biosystems™, Foster City, CA). Real-time qPCR was performed using StepOnePlus™ Real-Time PCR System (Applied Biosystems™, Foster City, CA). The expression level of each target gene was normalized against ACTB. Statistical significance of altered gene expression was determined using Student's *t*-test, where the significance was defined at *p*<0.05.

## Supporting Information

Figure S1 **The proteome maps of differentially expressed whole cell proteins in mock control and CHIKV-infected WRL-68 cells.**
(DOCX)

Table S1 **List of peptide sequences identified by MALDI-TOF/TOF MS.**
(DOCX)

Table S2 **Protein names and abbreviations used in STRING network analysis.**
(DOCX)

Table S3 **List of primer sequences used in real-time qPCR analysis.**
(DOCX)
