# Supplementary material for: Differential Proteome Analysis of Chikungunya Virus Infection on Host Cells
Source: PLoS One. 2013 Apr 10;8(4):e61444. doi: 10.1371/journal.pone.0061444 (PMC3622599; doi:10.1371/journal.pone.0061444)
Supplement: Figure S1 — The proteome maps of differentially expressed whole cell proteins in mock control and CHIKV-infected WRL-68 cells. (DOCX) [file pone.0061444.s001.docx]

**
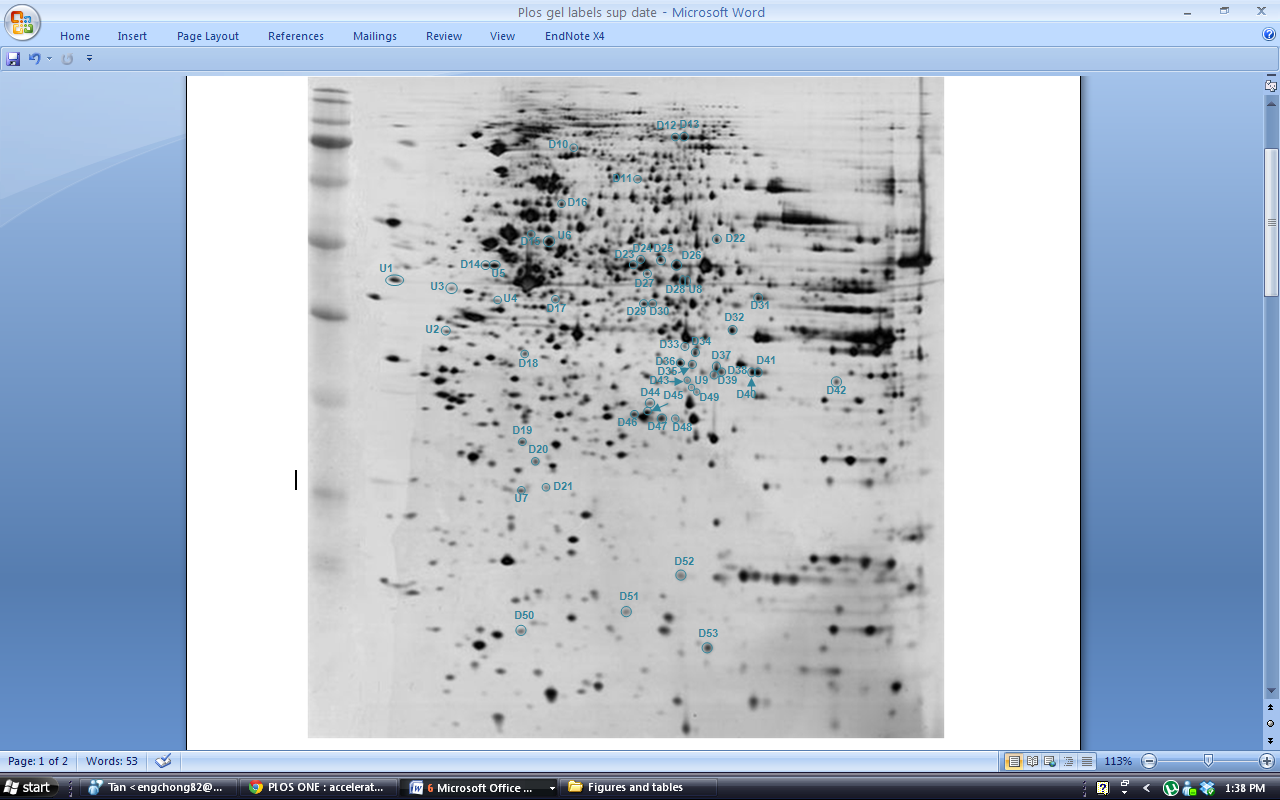

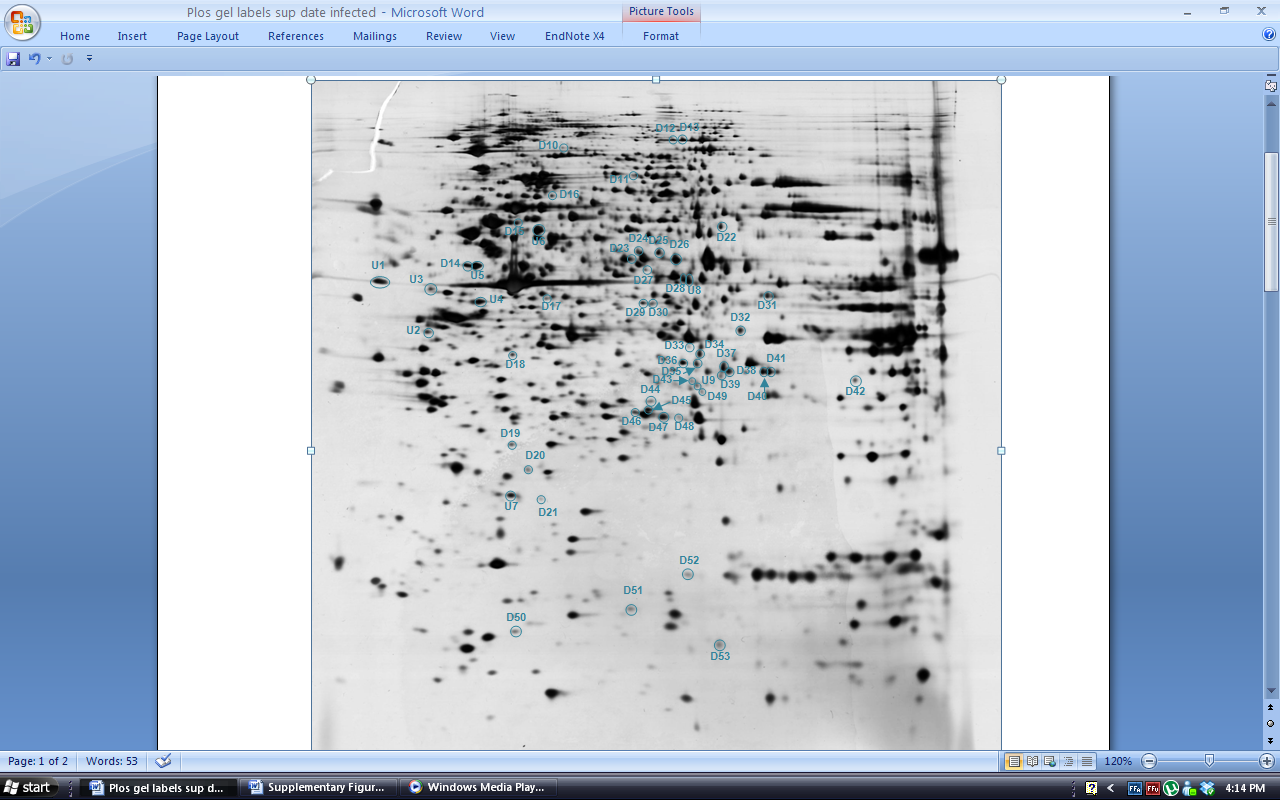
Supplementary Figure S1. The proteome maps of differentially expressed whole cell proteins in mock control and CHIKV-infected WRL-68 cells**

**kDa**

**180**

**135**

**100**

**75**

**63**

**48**

**35**

**23**

**17**

**11**

**pH Linear pH gradient pH**

**3 10**

**pH Linear pH gradient pH**

**3 10**

**Mock control CHIKV-infected (MOI 5.0)**
