# Supplementary material for: Differential Proteome Analysis of Chikungunya Virus Infection on Host Cells
Source: PLoS One. 2013 Apr 10;8(4):e61444. doi: 10.1371/journal.pone.0061444 (PMC3622599; doi:10.1371/journal.pone.0061444)
Supplement: Table S1 — List of peptide sequences identified by MALDI-TOF/TOF MS. (DOCX) [file pone.0061444.s002.docx]

**Supplementary Table S1. List of peptide sequences identified by MALDI-TOF/TOF MS**

| **Spot no.** | **Protein name** | **Peptides matched** | **Start-End** | **Miss** | **Ions** | **Peptides** |
| --- | --- | --- | --- | --- | --- | --- |
| U1 | Protein SET | 9 | 5-13  15-35  40-57  58-68  76-83  84-90  123-132  137-150  155-167 | 1  0  0  0  1  1  0  0  0 | 5  0  53  59  0  0  53  0  34 | K.RQSPLPPQK.K  K.KPRPPPALGPEETSASAGLPK.K  R.VEVTEFEDIK.S  R.LNEQASEEILK.V  K.LRQPFFQK.R  K.RSELIAK.I  R.VEVTEFEDIK.S  R.IDFYFDENPYFENK.V  K.EFHLNESGDPSSK.S |
| U2 | Nucleophosmin (B23) | 4 | 33-45  55-73  81-101  278-291 | 0  0  0  0 | 59  -  -  23 | K.VDNDENEHQLSLR.T  K.DELHIVEAEAMNYEGSPIK.V  K.MSVQPTVSLGGFEITPPVVLR.L  R.MTDQEAIQDLWQWR.K |
| U3 | Reticulocalbin-1 | 9 | 71-83  91-105  115-122  135-141  177-188  205-217  264-270  271-286  287-294 | 0  0  0  0  0  0  1  0  0 | -  117  18  19  -  -  31  -  54 | K.TFDQLTPDESKER.L  R.IDNDGDGFVTTEELK.T R.YIFDNVAK.V  K.ISWEEYK.Q  K.AADLNGDLTATR.E  K.EIVVLETLEDIDK.N  K.LDKDEIR.H  R.HWILPQDYDHAQAEAR.H  R.HLVYESDK.N |
| U4 | Heterogeneous nuclear ribonucleoproteins C1/C2 | 9 | 1-8  18-29  43-50  51-61  74-89  74-89  136-142  143-151  205-216 | 0  0  0  0  0  0  0  0  1 | -  15  -  51  (20)  53  -  46  58 | -.MASNVTNK.T  R.VFIGNLNTLVVK.K  K.IVGCSVHK.G  K.GFAFVQYVNER.N  R.MIAGQVLDINLAAEPK.V  R.MIAGQVLDINLAAEPK.V + Oxidation (M)  R.MYSYPAR.V + Oxidation (M)  R.VPPPPPIAR.A  K.QKVDSLLENLEK.I |
| U5 | Keratin, type I cytoskeletal 17 | 20 | 31-41  86-94  104-115  116-122  130-136  145-163  164-170  171-180  173-180  181-191  193-201  221-230  231-251  270-278  298-304  322-334  358-368  375-378  377-385  387-399 | 0  0  0  1  0  0  0  1  0  0  0  0  0  0  0  0  0  1  0  0 | -  1  -  4  29  -  25  9  -  25  -  39  95  48  42  72  -  25  38  79 | R.LSGGLGAGSCR.L + Carbamidomethyl (C)  K.ATMQNLNDR.L  R.ALEEANTELEVK.I  K.IRDWYQR.Q  R.DYSQYYR.T  K.ILTATVDNANILLQIDNAR.L  R.LAADDFR.T  R.TKFETEQALR.L  K.FETEQALR.L  R.LSVEADINGLR.R  R.VLDELTLAR.A  K.NHEEEMNALR.G  R.GQVGGEINVEMDAAPGVDLSR.I  K.DAEDWFFSK.T  K.SEISELR.R  K.ASLEGNLAETENR.Y  R.CEMEQQNQEYK.I + Carbamidomethyl (C)  K.TRLEQEIATYR.R  R.LEQEIATYR.R  R.LLEGEDAHLTQYK.K |
| U6 | Keratin, type II cytoskeletal 7 | 22 | 53-64  65-77  78-86  87-96  102-108  111-117  123-130  137-149  150-161  168-179  180-187  188-198  215-226  227-253  277-286  306-313  318-326  332-342  352-363  374-382  383-393  395-402 | 0  0  0  1  0  0  0  0  0  1  1  0  0  0  0  0  0  0  1  0  1  0 | 68  44  28  -  -  -  -  87  52  -  -  40  47  69  -  -  -  -  91  -  19  - | R.SAYGGPVGAGIR.E  R.EVTINQSLLAPLR.L  R.LDADPSLQR.V  R.VRQEESEQIK.T  K.FASFIDK.V  R.FLEQQNK.L  K.WTLLQEQK.S  R.LPDIFEAQIAGLR.G  R.GQLEALQVDGGR.L  R.TMQDVVEDFKNK.Y + Oxidation (M)  K.YEDEINRR.T  R.TAAENEFVVLK.K  K.VDALNDEINFLR.T  R.TLNETELTELQSQISDTSVVLSMDNSR.S  R.AEAEAWYQTK.F  R.NEISEMNR.A  R.LQAEIDNIK.N  K.LEAAIAEAEER.G  R.AKQEELEAALQR.A  R.EYQELMSVK.L  K.LALDIEIATYR.K  K.LLEGEESR.L |
| U7 | Chromobox protein homolog 3 | 4 | 21-34  142-154  160-171  172-183 | 1  1  0  0 | -  -  73  - | K.KVEEAEPEEFVVEK.V  K.WKDSDEADLVLAK.E  K.CPQIVIAFYEER.L + Carbamidomethyl (C)  R.LTWHSCPEDEAQ.- + Carbamidomethyl (C) |
| U8 | Pyruvate dehydrogenase E1 component subunit alpha, mitochondrial | 7 | 46-58  133-141  216-226  246-253  254-263  268-274  278-288 | 0  0  0  0  0  0  0 | 61  12  -  -  -  -  21 | R.LEEGPPVTTVLTR.E  R.EILAELTGR.K  K.LPCIFICENNR.Y + 2 Carbamidomethyl (C)  R.GDFIPGLR.V  R.VDGMDILCVR.E + Carbamidomethyl (C)  R.FAAAYCR.S + Carbamidomethyl (C)  K.GPILMELQTYR.Y |
| D10 | Spartin | 4 | 63-75  335-341  342-375  616-636 | 0  0  0  0 | -  -  71  - | K.ESEHTGPGWESAR.Q R.LQANWNR.A  R.AEEENEFQIPGR.T  K.TATQTGHTLLEDYQIVDNSQR.E |
| D11 | Phosphoglucomutase-2 | 13 | 15-24  38-48  55-63  73-87  100-108  176-190  248-268  269-282  317-327  391-403  467-478  479-491  513-519 | 0  1  0  0  0  0  1  0  0  0  0  0  0 | 30  -  -  -  -  -  -  -  -  57  63  -  - | R.LDQETAQWLR.W  R.LIAEGNKEELR.K  R.MEFGTAGLR.A  R.MNDLTIIQTTQGFCR.Y + Carbamidomethyl (C)  K.GIVISFDAR.A  K.VYWDNGAQIISPHDK.G  K.VKFVHTSVHGVGHSFVQSAFK.A  K.AFDLVPPEAVPEQK.D  R.IVLANDPDADR.L  K.EGFHFEETLTGFK.W  K.AIYVEYGYHITK.A  K.ASYFICHDQETIK.K + Carbamidomethyl (C)  K.FEISAIR.D |
| D12 | Elongation factor-2 | 27 | 2-10  21-32  163-180  240-249  253-259  273-283  288-299  300-308  329-337  369-386  401-409  416-426  440-449  450-456  482-495  499-506  573-580  606-625  626-631  639-647  648-667  677-688  689-698  717-726  728-739  768-785  786-801 | 0  0  0  0  1  1  0  0  1  0  1  0  0  0  0  0  0  1  1  0  0  0  0  0  0  0  0 | 1  -  -  -  -  -  59  -  -  -  -  -  51  -  31  9  -  63  -  43  -  -  -  28  28  -  5 | M.VNFTVDQIR.A  R.NMSVIAHVDHGK.S + Oxidation (M)  R.ALLELQLEPEELYQTFQR.I  K.GEGQLGPAER.A  K.VEDMMKK.L + 2 Oxidation (M)  K.FSKSATSPEGK.K  R.TFCQLILDPIFK.V + Carbamidomethyl (C)  K.VFDAIMNFK.K  K.DKEGKPLLK.A  R.CELLYEGPPDDEAAMGIK.S + Carbamidomethyl (C)  K.MVPTSDKGR.F + Oxidation (M)  R.VFSGLVSTGLK.V  K.EDLYLKPIQR.T  R.TILMMGR.Y  K.TGTITTFEHAHNMR.V  K.FSVSPVVR.V  K.SDPVVSYR.E  K.ARPFPDGLAEDIDKGEVSAR.Q  K.YEWDVAEAR.K  R.QELKQR.A  R.KIWCFGPDGTGPNILTDITK.G + Carbamidomethyl (C)  K.DSVVAGFQWATK.E  K.EGALCEENMR.G + Carbamidomethyl (C)  R.GGGQIIPTAR.R  R.CLYASVLTAQPR.L + Carbamidomethyl (C)  R.GHVFEESQVAGTPMFVVK.A  K.AYLPVNESFGFTADLR.S |
| D13 | Elongation factor-2 | 11 | 2-10  121-144  163-180  273-283  288-308  482-495  606-625  639-647  728-739  768-785  786-801 | 0  0  0  1  1  0  1  0  0  0  0 | 35  29  -  -  -  -  65  46  -  41  78 | M.VNFTVDQIR.A  R.VTDGALVVVDCVSGVCVQTETVLR.Q + 2 Carbamidomethyl (C)  R.ALLELQLEPEELYQTFQR.I  K.FSKSATSPEGK.K  R.TFCQLILDPIFKVFDAIMNFK.K + Carbamidomethyl (C); Oxidation (M)  K.TGTITTFEHAHNMR.V  K.ARPFPDGLAEDIDKGEVSAR.Q  K.YEWDVAEAR.K  R.CLYASVLTAQPR.L + Carbamidomethyl (C)  R.GHVFEESQVAGTPMFVVK.A  K.AYLPVNESFGFTADLR.S |
| D14 | Gamma-enolase | 13 | 6-15  16-28  33-50  90-103  121-132  184-193  240-253  270-285  344-358  359-372  407-412  413-422  423-429 | 1  0  0  0  1  0  0  0  0  0  0  0  0 | -  65  -  -  -  66  -  -  -  -  15  -  - | K.IWAREILDSR.G  R.GNPTVEVDLYTAK.G  R.AAVPSGASTGIYEALELR.D  K.LDNLMLELDGTENK.S  K.AGAAERELPLYR.H  R.LGAEVYHTLK.G  K.IVIGMDVAASEFYR.D  R.YITGDQLGALYQDFVR.D  K.VNQIGSVTEAIQACK.L + Carbamidomethyl (C)  K.LAQENGWGVMVSHR.S  K.YNQLMR.I  R.IEEELGDEAR.F  R.FAGHNFR.N |
| D15 | Hydroxymethylglutaryl-CoA synthase, cytoplasmic | 8 | 2-15  80-89  220-231  240-246  278-289  292-305  306-313  416-428 | 0  0  0  0  0  0  0  0 | -  13  -  -  -  -  6  - | M.PGSLPLNAEACWPK.D + Carbamidomethyl (C)  R.NNLSYDCIGR.L + Carbamidomethyl (C)  K.LSIQCYLSALDR.C + Carbamidomethyl (C)  K.IHAQWQK.E  R.MLLNDFLNDQNR.D  K.NSIYSGLEAFGDVK.L  K.LEDTYFDR.D  R.TGVAPDVFAENMK.L |
| D16 | Copine-1 | 3 | 61-67  158-166  479-487 | 0  0  0 | 16  52  58 | K.TLQLEYR.F  R.DIVQFVPYR.R  K.SDPFLEFFR.Q |
| D18 | Spermidine synthase | 6 | 48-55  58-74  97-109  149-161  187-194  286-295 | 0  0  0  0  0  0 | 40  42  50  -  -  33 | R.YQDILVFR.S  K.TYGNVLVLDGVIQCTER.D + Carbamidomethyl (C)  K.VLIIGGGDGGVLR.E  K.LTLHVGDGFEFMK.Q  K.ESYYQLMK.T  R.AAFVLPEFAR.K |
| D19 | Ubiquitin-conjugating enzyme E2 N | 6 | 29-40  41-55  62-72  98-107  147-164  166-176 | 0  0  0  0  0  0 | 26  14  35  -  91  - | K.VDLVDENFTELR.G  R.GEIAGPPDTPYEGGR.Y  K.IPETYPFNPPK.V  K.DQWAAAMTLR.T  R.LWAHVYAGAPVSSPEYTK.K  K.IENLCAMGFDR.N + Carbamidomethyl (C) |
| D20 | Inosine triphosphate pyrophosphatase | 3 | 40-56  95-110  111-130 | 0  0  0 | 58  -  21 | K.IDLPEYQGEPDEISIQK.C  K.LKPEGLHQLLAGFEDK.S  K.SAYALCTFALSTGDPSQPVR.L + Carbamidomethyl (C) |
| D21 | Adenine phosphoribosyltransferase | 7 | 15-27  28-40  58-67  92-107  115-122  123-145  146-163 | 0  1  0  0  0  0  0 | 60  23  34  66  -  -  - | R.SFPDFPTPGVVFR.D  R.DISPVLKDPASFR.A  R.IDYIAGLDSR.G  K.LPGPTLWASYSLEYGK.A  K.DALEPGQR.V  R.VVVVDDLLATGGTMNAACELLGR.L + Carbamidomethyl (C)  R.LQAEVLECVSLVELTSLK.G + Carbamidomethyl (C) |
| D22 | Nicotinamide phosphoribosyltransferase | 8 | 33-40  108-117  118-127  190-196  290-296  303-323  343-349  470-477 | 0  0  0  0  0  0  0  0 | 15  -  34  23  24  -  -  21 | K.VYSYFECR.E + Carbamidomethyl (C)  K.YDGHLPIEIK.A  K.AVPEGFVIPR.G  K.LHDFGYR.G  K.IWGEDLR.H  R.STQAPLIIRPDSGNPLDTVLK.V  K.LLPPYLR.V   \|  \| \| --- \| \|  \| \|  \|   K.SYSFDEIR.K |
| D23 | Rab GDP dissociation inhibitor beta | 14 | 56-68  69-79  90-98  143-156  194-208  211-218  222-240  279-288  291-299  310-328  329-348  365-379  391-402  424-436 | 1  0  0  1  0  0  0  0  1  0  1  0  0  1 | 22  19  -  31  67  6  -  -  -  102  -  52  56  - | R.FKIPGSPPESMGR.G  R.GRDWNVDLIPK.F  K.MLLYTEVTR.Y  K.FLVYVANFDEKDPR.T  R.TDDYLDQPCYETINR.I + Carbamidomethyl (C)  K.LYSESLAR.Y  K.SPYLYPLYGLGELPQGFAR.L   \| K.QLICDPSYVK.D + Carbamidomethyl (C) \| \| --- \| \|  \|   R.VEKVGQVIR.V  K.NTNDANSCQIIIPQNQVNR.K + Carbamidomethyl (C)  R.KSDIYVCMISFAHNVAAQGK.Y + Carbamidomethyl (C)  K.EIRPALELLEPIEQK.F  K.DLGTESQIFISR.T  R.MTGSEFDFEEMKR.K |
| D24 | Rab GDP dissociation inhibitor beta | 11 | 56-68  69-79  143-156  194-208  211-218  222-240  291-299  310-328  329-348  365-379  391-402 | 1  1  1  0  0  0  1  0  1  0  0 | -  -  -  38  -  -  -  -  -  7  42 | R.FKIPGSPPESMGR.G  R.GRDWNVDLIPK.F  K.FLVYVANFDEKDPR.T  R.TDDYLDQPCYETINR.I + Carbamidomethyl (C)  K.LYSESLAR.Y  K.SPYLYPLYGLGELPQGFAR.L  R.VEKVGQVIR.V  K.NTNDANSCQIIIPQNQVNR.K + Carbamidomethyl (C)  R.KSDIYVCMISFAHNVAAQGK.Y + Carbamidomethyl (C)  K.EIRPALELLEPIEQK.F  K.DLGTESQIFISR.T |
| D25 | La ribonucleoprotein | 10 | 55-60  92-105  135-143  167-174  236-246  261-266  270-276  288-297  300-312  318-328 | 1  0  0  0  0  0  0  0  0  0 | -  13  16  -  15  3  10  -  -  - | K.FNRLNR.L  R.SPSKPLPEVTDEYK.N  K.GQVLNIQMR.R  K.FVETPGQK.Y  K.FSGDLDDQTCR.E + Carbamidomethyl (C)  K.WIDFVR.G  K.EGIILFK.E  K.DANNGNLQLR.N  K.EVTWEVLEGEVEK.E  K.IIEDQQESLNK.W |
| D26 | Alpha-enolase | 9 | 16-28  33-50  163-179  184-193  240-253  270-281  307-326  359-372  407-412 | 0  0  0  0  0  0  0  0  0 | -  -  -  -  9  36  13  -  - | R.GNPTVEVDLFTSK.G  R.AAVPSGASTGIYEALELR.D  K.LAMQEFMILPVGAANFR.E  R.IGAEVYHNLK.N  K.VVIGMDVAASEFFR.S  R.YISPDQLADLYK.S  K.FTASAGIQVVGDDLTVTNPK.R  K.LAQANGWGVMVSHR.S  K.YNQLLR.I |
| D27 | Adenylosuccinate synthetase isozyme 2 | 16 | 30-43  46-59  119-125  133-151  181-194  197-203  204-219  233-247  304-324  327-334  338-347  380-397  404-416  420-430  431-441  450-456 | 0  0  0  0  0  0  0  0  0  0  0  0  0  0  0  0 | -  13  -  86  43  -  -  56  5  -  -  78  50  25  -  - | R.VTVVLGAQWGDEGK.G  K.VVDLLAQDADIVCR.C + Carbamidomethyl (C)  K.GLEGWEK.R  R.AHIVFDFHQAADGIQEQQR.Q  R.MCDLVSDFDGFSER.F + Carbamidomethyl (C)  K.VLANQYK.S  K.SIYPTLEIDIEGELQK.L  R.DGVYFLYEALHGPPK.K  R.VGIGAFPTEQDNEIGELLQTR.G  R.EFGVTTGR.K  R.CGWLDLVLLK.Y  K.LDGEIIPHIPANQEVLNK.V  K.TLPGWNTDISNAR.A  K.ELPVNAQNYVR.F  R.FIEDELQIPVK.W  R.ESMIQLF.- + Oxidation (M) |
| D28 | Isocitrate dehydrogenase, cytoplasmic | 10 | 5-20  21-27  101-109  120-132  133-140  141-151  204-212  244-249  261-270  389-400 | 0  0  0  0  0  0  0  0  0  0 | 37  10  -  3  18  -  -  -  56  - | K.ISGGSVVEMQGDEMTR.I  R.IIWELIK.E  R.NILGGTVFR.E  R.LVSGWVKPIIIGR.H  R.HAYGDQYR.A  R.ATDFVVPGPGK.V  K.GWPLYLSTK.N  K.IWYEHR.L  K.SEGGFIWACK.N + Carbamidomethyl (C)  R.SDYLNTFEFMDK.L |
| D29 | Eukaryotic translation initiation factor 3 subunit H | 4 | 181-188  292-303  304-313  332-340 | 0  0  0  0 | 23  45  35  30 | K.DFSPEALK.K  R.GEPPLPEEDLSK.L  K.LFKPPQPPAR.M  K.EFTAQNLGK.L |
| D30 | Poly(rC)-binding protein1 (hnRNP E1) | 7 | 47-57  102-115  125-144  145-160  178-200  298-306  315-325 | 0  0  0  0  0  0  0 | -  48  25  -  -  -  - | R.INISEGNCPER.I + Carbamidomethyl (C)  R.LVVPATQCGSLIGK.G + Carbamidomethyl (C)  R.ESTGAQVQVAGDMLPNSTER.A  R.AITIAGVPQSVTECVK.Q + Carbamidomethyl (C)  R.VMTIPYQPMPASSPVICAGGQDR.C + Carbamidomethyl (C)  R.QGANINEIR.Q  K.IANPVEGSSGR.Q |
| D31 | Phosphoserine aminotransferase | 5 | 52-61  118-127  191-200  214-222  343-356 | 0  0  0  0  0 | -  15  38  -  - | K.IINNTENLVR.E  K.FGTINIVHPK.L  K.FGVIFAGAQK.N  R.DDLLGFALR.E  R.ASLYNAVTIEDVQK.L |
| D32 | Aldo-keto reductase family 1 member C2 | 9 | 10-31  76-84  85-101  137-153  162-171  184-200  210-223  251-258  279-294 | 0  1  0  0  0  0  0  0  0 | 79  -  6  -  -  20  -  -  88 | K.LNDGHFMPVLGFGTYAPAEVPK.S  K.REDIFYTSK.L  K.LWSNSHRPELVRPALER.S  K.ILFDTVDLCATWEAMEK.C + Carbamidomethyl (C)  K.YKPVCNQVECHPYFNQR.K + 2 Carbamidomethyl (C)  K.SIGVSNFNHR.L  K.DIVLVAYSALGSHR.E  R.TPALIALR.Y  R.QNVQVFEFQLTSEEMK.A |
| D33 | Pirin | 8 | 6-14  27-34  85-97  126-135  145-160  167-175  212-231  236-247 | 1  0  0  0  0  0  0  0 | -  -  1  -  40  -  -  18 | K.KVTLSVLSR.E  R.SIGRPELK.N  K.MNPGDLQWMTAGR.G  K.MVEPQYQELK.S  K.DGVTVAVISGEALGIK.S  R.TPTLYLDFK.L  K.IEPHHTAVLGEGDSVQVENK.D  R.SHFVLIAGEPLR.E |
| D34 | Ribose-phosphate pyrophosphokinase1 | 9 | 6-18  35-49  85-96  157-163  185-194  205-212  215-235  244-260  261-280 | 0  0  0  0  0  0  0  0  0 | -  14  34  9  -  -  -  -  - | K.IFSGSSHQDLSQK.I  K.FSNQETCVEIGESVR.G + Carbamidomethyl (C)  R.VTAVIPCFPYAR.Q + Carbamidomethyl (C)  R.ENISEWR.N  R.LNVDFALIHK.E  R.MVLVGDVK.D  R.VAILVDDMADTCGTICHAADK.L + 2 Carbamidomethyl (C)  R.VYAILTHGIFSGPAISR.I  R.INNACFEAVVVTNTIPQEDK.M + Carbamidomethyl (C) |
| D35 | Glucosamine-6-phosphate isomerase 1 | 7 | 24-33  52-57 68-79  161-172  173-180  181-197  235-248 | 0  0  0  0  0  0  0 | 23  -  -  2  24  -  - | R.IIQFNPGPEK.Y  K.LIEYYK.N  K.TFNMDEYVGLPR.D  K.TLAMDTILANAR.F  R.FFDGELTK.V  K.VPTMALTVGVGTVMDAR.E  R.TVFVCDEDATLELK.V + Carbamidomethyl (C) |
| D36 | S-formylglutathione hydrolase | 7 | 18-29  32-39  65-86  143-161  187-198  201-209  247-253 | 0  0  0  0  0  0  1 | -  -  -  -  5  -  - | K.VFEHDSVELNCK.M + Carbamidomethyl (C)  K.FAVYLPPK.A  K.SGYHQSASEHGLVVIAPDTSPR.G  R.MSIFGHSMGGHGALICALK.N + Carbamidomethyl (C); Oxidation (M)  K.AFSGYLGTDQSK.W  K.AYDATHLVK.S  K.KIPVVFR.L |
| D37 | Actin-related protein 2/3 complex subunit 2 (p34-ARC) | 12 | 1-9  66-77  107-117  128-136  128-141  148-158  180-186  191-203  211-230  238-248  249-256  270-279 | 0  0  0  0  1  1  0  0  0  0  0  1 | 4  -  -  33  22  -  21  33  -  -  -  - | -.MILLEVNNR.I  K.ELQAHGADELLK.R  K.DSIVHQAGMLK.R  K.YFQFQEEGK.E  K.YFQFQEEGKEGENR.A  R.DDETMYVESKK.D + Oxidation (M)  K.VFMQEFK.E  R.ASHTAPQVLFSHR.E  K.DTDAAVGDNIGYITFVLFPR.H  R.DNTINLIHTFR.D  R.DYLHYHIK.C  K.TSDFLKVLNR.A |
| D38 | Electron transfer flavoprotein subunit alpha, mitochondrial | 5 | 102-117  140-146  188-203  233-249  250-268 | 0  0  0  0  0 | 72  23  -  11  - | K.QFNYTHICAGASAFGK.N + Carbamidomethyl (C)  K.SPDTFVR.T  K.ASSTSPVEISEWLDQK.L  K.LLYDLADQLHAAVGASR.A  R.AAVDAGFVPNDMQVGQTGK.I |
| D39 | Guanine nucleotide-binding protein subunit beta-2-like 1 | 5 | 48-57  89-99  140-155  246-257  309-317 | 0  0  0  0  0 | 37  -  -  -  8 | R.DETNYGIPQR.A  R.LWDLTTGTTTR.R K.YTVQDESHSEWVSCVR.F + Carbamidomethyl (C)  R.YWLCAATGPSIK.I  R.VWQVTIGTR.- |
| D40 | Guanine nucleotide-binding protein subunit beta-2-like 1 | 15 | 48-57  89-99  107-118  131-139  140-155  156-172  176-183  186-212  213-225  226-245  246-257  258-264  265-271  272-280  309-317 | 0  0  0  0  0  0  0  0  0  0  0  0  0  0  0 | 30  34  57  38  29  -  -  -  -  -  43  30  -  -  18 | R.DETNYGIPQR.A  R.LWDLTTGTTTR.R  K.DVLSVAFSSDNR.Q  K.LWNTLGVCK.Y + Carbamidomethyl (C)  K.YTVQDESHSEWVSCVR.F + Carbamidomethyl (C)  R.FSPNSSNPIIVSCGWDK.L + Carbamidomethyl (C)  K.VWNLANCK.L + Carbamidomethyl (C)  K.TNHIGHTGYLNTVTVSPDGSLCASGGK.D + Carbamidomethyl (C)  K.DGQAMLWDLNEGK.H  K.HLYTLDGGDIINALCFSPNR.Y + Carbamidomethyl (C)  R.YWLCAATGPSIK.I  K.IWDLEGK.I  K.IIVDELK.Q  K.QEVISTSSK.A  R.VWQVTIGTR.- |
| D41 | Guanine nucleotide-binding protein subunit beta-2-like 1 | 16 | 48-57  89-99  107-118  131-139  140-155  156-172  176-183  186-212  213-225  226-245  246-257  258-264  265-271  272-280  281-308  309-317 | 0  0  0  0  0  0  0  0  0  0  0  0  0  0  0  0 | 37  40  18  43  85  57  32  -  71  -  39  16  -  -  14  - | R.DETNYGIPQR.A  R.LWDLTTGTTTR.R  K.DVLSVAFSSDNR.Q  K.LWNTLGVCK.Y + Carbamidomethyl (C)  K.YTVQDESHSEWVSCVR.F + Carbamidomethyl (C)  R.FSPNSSNPIIVSCGWDK.L + Carbamidomethyl (C)  K.VWNLANCK.L + Carbamidomethyl (C)  K.TNHIGHTGYLNTVTVSPDGSLCASGGK.D + Carbamidomethyl (C)  K.DGQAMLWDLNEGK.H  K.HLYTLDGGDIINALCFSPNR.Y + Carbamidomethyl (C)  R.YWLCAATGPSIK.I + Carbamidomethyl (C)  K.IWDLEGK.I  K.IIVDELK.Q  K.QEVISTSSK.A  K.AEPPQCTSLAWSADGQTLFAGYTDNLVR.V + Carbamidomethyl (C)  R.VWQVTIGTR.- |
| D42 | Cyclin-dependent kinase 1 | 13 | 10-20  25-33  37-50  60-75  90-106 128-143  144-151  159-170  171-180  201-215  219-238  267-275  280-295 | 0  0  0  0  0  1  0  0  0  0  0  1  0 | -  -  46  -  -  -  25  38  16  37  8  -  3 | K.IGEGTYGVVYK.G  K.TTGQVVAMK.K + Oxidation (M)  R.LESEEEGVPSTAIR.E  R.HPNIVSLQDVLMQDSR.L  K.YLDSIPPGQYMDSSLVK.S  R.DLKPQNLLIDDK.G  K.LADFGLAR.A  R.VYTHEVVTLWYR.S  R.SPEVLLGSAR.Y  K.KPLFHGDSEIDQLFR.I  R.ALGTPNNEVWPEVESLQDYK.N  K.MLIYDPAKR.I  K.MALNHPYFNDLDNQIK.K |
| D43 | Translation initiation factor eIF-2B subunit alpha | 8 | 16-28  37-46  75-88  111-120  121-132  148-162  221-234  238-151 | 0  1  0  0  0  0  0  1 | -  32  -  -  23  -  -  - | K.SQMKEDPDMASAVAAIR.T  R.DKGETIQGLR.A  R.FISLASLEYSDYSK.C  K.IADLCHTFIK.D + Carbamidomethyl (C)  K.DGATILTHAYSR.V  R.FSVYVTESQPDLSGK.K  K.AQNKPFYVVAESFK.F  R.LFPLNQQDVPDKFK.Y |
| D44 | Phosphoglycerate mutase 1 | 8 | 1-10  11-21  22-39  47-61  91-100  118-138  142-157  181-191 | 1  0  0  0  0  0  0  0 | -  72  -  63  68  -  61  34 | -.MAAYKLVLIR.H + Oxidation (M)  R.HGESAWNLENR.F  R.FSGWYDADLSPAGHEEAK.R  R.DAGYEFDICFTSVQK.R + Carbamidomethyl (C)  R.SYDVPPPPMEPDHPFYSNISK.D  R.HYGGLTGLNK.A  R.YADLTEDQLPSCESLK.D + Carbamidomethyl (C)  R.VLIAAHGNSLR.G |
| D45 | Proteasome subunit alpha type-6 | 10 | 2-11  12-21  22-30  31-43  60-71  72-88  96-102  105-116  154-164  229-245 | 1  0  0  0  0  0  0  0  0  0 | -  14  -  -  -  -  -  58  47  1 | M.SRGSSAGFDR.H  R.HITIFSPEGR.L  R.LYQVEYAFK.A  K.AINQGGLTSVAVR.G  K.LLDSSTVTHLFK.I  K.ITENIGCVMTGMTADSR.S + Carbamidomethyl (C)  R.YEAANWK.Y  K.YGYEIPVDMLCK.R + Carbamidomethyl (C)  K.CDPAGYYCGFK.A + 2 Carbamidomethyl (C)  R.ILTEAEIDAHLVALAER.D |
| D46 | Isopentyl-diphosphate Delta-isomerase 1 | 2 | 53-64  114-133 | 0  0 | 45  73 | R.AFSVFLFNTENK.L  K.AELGIPLEEVPPEEINYLTR.I |
| D47 | Triosephosphate isomerase | 10 | 7-14  34-53  60-69  86-99  100-113  114-131  161-175  176-188  195-206  207-219 | 0  0  0  0  1  0  0  0  0  0 | 2  35  -  16  -  -  -  -  -  - | K.FFVGGNWK.M  K.VPADTEVVCAPPTAYIDFAR.Q + Carbamidomethyl (C)  K.IAVAAQNCYK.V + Carbamidomethyl (C)  K.DCGATWVVLGHSER.R + Carbamidomethyl (C)  R.HVFGESDELIGQK.V  K.VAHALAEGLGVIACIGEK.L + Carbamidomethyl (C)  K.VVLAYEPVWAIGTGK.T  K.TATPQQAQEVHEK.L  K.SNVSDAVAQSTR.I  R.IIYGGSVTGATCK.E + Carbamidomethyl (C) |
| D48 | Triosephosphate isomerase | 5 | 7-14  34-53  86-99  100-113  161-175 | 0  0  0  0  0 | 14  63  21  -  - | K.FFVGGNWK.M  K.VPADTEVVCAPPTAYIDFAR.Q + Carbamidomethyl (C)  K.DCGATWVVLGHSER.R + Carbamidomethyl (C)  R.HVFGESDELIGQK.V  K.VVLAYEPVWAIGTGK.T |
| D49 | S-methyl-5-thioadenosine phosphorylase | 11 | 12-29  33-49  52-60  64-71  72-82  83-99  134-147  167-176  181-187  226-235  272-282 | 0  0  0  0  0  0  0  0  0  0  0 | 29  -  11  -  -  24  51  10  -  42  27 | K.IGIIGGTGLDDPEILEGR.T  K.YVDTPFGKPSDALILGK.I  K.NVDCILLAR.H + Carbamidomethyl (C)  R.QHTIMPSK.V  K.VNYQANIWALK.E  K.EEGCTHVIVTTACGSLR.E + 2 Carbamidomethyl (C)  R.GVCHIPMAEPFCPK.T + 2 Carbamidomethyl (C)  K.GTMVTIEGPR.F  R.AESFMFR.T  K.EHEEAVSVDR.V  K.NMAQFSVLLPR.H |
| D50 | Thioredoxin-like protein 5 | 2 | 4-17  41-54 | 0  0 | 38  48 | R.YEEVSVSGFEEFHR.A  K.SWCPDCVQAEPVVR.E + 2 Carbamidomethyl (C) |
| D51 | Fatty-acid binding protein, epidermal | 7 | 18-24  25-33  35-50  35-55  62-72  73-81  116-129 | 0  0  0  1  0  0  0 | -  10  -  -  3  -  36 | K.GFDEYMK.E  K.ELGVGIALR.K  K.MGAMAKPDCIITCDGK.N + Carbamidomethyl (C)  K.MGAMAKPDCIITCDGKNLTIK.T + Oxidation (M)  K.TTQFSCTLGEK.F + Carbamidomethyl (C)  K.FEETTADGR.K  K.LVVECVMNNVTCTR.I + 2 Carbamidomethyl (C) |
| D52 | Peptidyl-prolyl cis-trans isomerase A (Cyclophilin A) | 8 | 20-28  56-69  56-69  83-91  119-125  156-165  132-144  134-144 | 0  0  0  0  0  0  1  0 | 31  (9)  29  -  -  16  -  - | R.VSFELFADK.V  R.IIPGFMCQGGDFTR.H + Carbamidomethyl (C)  R.IIPGFMCQGGDFTR.H + Carbamidomethyl (C); Oxidation (M)  K.FEDENFILK.H  K.TEWLDGK.H  K.ITIADCGQLE.- + Carbamidomethyl (C)  K.VKEGMNIVEAMER.F  K.EGMNIVEAMER.F + Oxidation (M) |
