# Supplementary material for: Differential Proteome Analysis of Chikungunya Virus Infection on Host Cells
Source: PLoS One. 2013 Apr 10;8(4):e61444. doi: 10.1371/journal.pone.0061444 (PMC3622599; doi:10.1371/journal.pone.0061444)
Supplement: Table S2 — Protein names and abbreviations used in STRING network analysis. (DOCX) [file pone.0061444.s003.docx]

**Supplementary Table S2. Protein names and gene symbols used in STRING network analysis**

| **Protein name** | **Gene name^a^** |
| --- | --- |
| Protein SET | SET |
| Nucleophosmin (B23) | NPM1 |
| Reticulocalbin-1 | RCN1 |
| Heterogeneous nuclear ribonucleoproteins C1/C2 | HNRNPC |
| Keratin, type I cytoskeletal 17 | KRT17 |
| Keratin, type II cytoskeletal 7 | KRT7 |
| Chromobox protein homolog 3 | CBX3 |
| Pyruvate dehydrogenase E1 component subunit alpha, mitochondrial | PDHA1 |
| Spartin | SPG20 |
| Phosphoglucomutase-2 | PGM2 |
| Elongation factor-2 | EEF2 |
| Gamma-enolase | ENO2 |
| Hydroxymethylglutaryl-CoA synthase, cytoplasmic | HMGCS1 |
| Copine-1 | CPNE1 |
| Spermidine synthase | SRM |
| Ubiquitin-conjugating enzyme E2 N | UBE2N |
| Inosine triphosphate pyrophosphatase | ITPA |
| Adenine phosphoribosyltransferase | APRT |
| Nicotinamide phosphoribosyltransferase | NAMPT |
| Rab GDP dissociation inhibitor beta | GDI2 |
| La ribonucleoprotein | SSB |
| Alpha-enolase | ENO1 |
| Adenylosuccinate synthetase isozyme 2 | ADSS |
| Isocitrate dehydrogenase [NADP] cytoplasmic | IDH1 |
| Eukaryotic translation initiation factor 3 subunit H | EIF3H |
| Poly(rC)-binding protein1 (hnRNP E1) | PCBP1 |
| Phosphoserine aminotransferase | PSAT1 |
| Aldo-keto reductase family 1 member C2 | AKR1C2 |
| Pirin | PIR |
| Ribose-phosphate pyrophosphokinase1 | PRPS1 |
| Glucosamine-6-phosphate isomerase 1 | GNPDA1 |
| S-formylglutathione hydrolase | ESD |
| Actin-related protein 2/3 complex subunit 2 (p34-ARC) | ARPC2 |
| Electron transfer flavoprotein subunit alpha, mitochondrial | ETFA |
| Guanine nucleotide-binding protein subunit beta-2-like 1 | GNB2L1 |
| Cyclin-dependent kinase 1 | CDK1 |
| Translation initiation factor eIF-2B subunit alpha | EIF2B1 |
| Phosphoglycerate mutase 1 | PGAM1 |
| Proteasome subunit alpha type-6 | PSMA6 |
| Isopentyl-diphosphate Delta-isomerase 1 | IDI1 |
| Triosephosphate isomerase | TPI1 |
| S-methyl-5-thioadenosine phosphorylase | MTAP |
| Thioredoxin-like protein 5 | TXNDC17 |
| Fatty-acid binding protein, epidermal | FABP5 |
| Peptidyl-prolyl cis-trans isomerase A (Cyclophilin A) | PPIA |
| Proteasome subunit alpha type-1 | PSMA1 |
| Proteasome subunit alpha type-2 | PSMA2 |
| Proteasome subunit alpha type-3 | PSMA3 |
| Proteasome subunit alpha type-4 | PSMA4 |
| Proteasome subunit beta type-1 | PSMB1 |
| Proteasome subunit beta type-2 | PSMB2 |
| Proteasome subunit beta type-3 | PSMB3 |
| Proteasome subunit beta type-4 | PSMB4 |
| Proteasome subunit beta type-5 | PSMB5 |
| Cyclin-dependent kinase 2 | CDK2 |
| Cyclin-dependent kinase inhibitor 1B | CDKN1B |
| Cyclin A1 | CCNA1 |
| Cyclin A2 | CCNA2 |
| Cyclin B1 | CCNB1 |
| Cyclin B2 | CCNB2 |
| Actin related protein 2/3 complex subunit 3 | APRC3 |
| Actin related protein 2/3 complex subunit 4 (20 kDa) | ARPC20 |
| Ubiquitin-conjugating enzyme E2 variant 2 | UBE2V2 |
| Electron transfer flavoprotein, beta polypeptide | ETFB |
| Pyruvate dehydrogenase beta | PDHB |
