# Supplementary material for: Differential Proteome Analysis of Chikungunya Virus Infection on Host Cells
Source: PLoS One. 2013 Apr 10;8(4):e61444. doi: 10.1371/journal.pone.0061444 (PMC3622599; doi:10.1371/journal.pone.0061444)
Supplement: Table S3 — List of primer sequences used in real-time qPCR analysis. (DOCX) [file pone.0061444.s004.docx]

**Supplementary Table S3. List of primer sequences used in real-time qPCR analysis**

| **Gene name^a^** | **Primer sequences** |
| --- | --- |
| UBE2N_F258 | 5’- CGTCACAGGGGCTATTTGTT-3’ |
| UBE2N_R379 | 5’- CCTGTGGATCATCTGGCTCT-3’ |
| PSMA6_F100 | 5’- CAGGGTGGCCTTACATCAGT-3’ |
| PSMA6_R249 | 5’- CATTCCGGTCATCACACAAC-3’ |
| SET_F576 | 5’- GAAGAGGCAGCATGAGGAAC-3’ |
| SET_R720 | 5’- ATCATCCATATCGGGAACCA-3’ |
| GNB2L1_F509 | 5’- GGGACAAGCTGGTCAAGGTA-3’ |
| GNB2L1_R616 | 5’- GGGATCCATCTGGAGAGACA-3’ |
| CDK1_F123 | 5’- GGAAGGGGTTCCTAGTACTGC-3’ |
| CDK1_R223 | 5’- TGGAATCCTGCATAAGCACA-3’ |
| PDHA1_F473 | 5’- AGAACTTCTACGGGGGCAAT-3’ |
| PDHA1_R616 | 5’- CGAATATCTGGCCCTGGTTA-3’ |
| ENO1_F765 | 5’- CTCCGTGACCGAGTCTCTTC-3’ |
| ENO1_R908 | 5’- CCAGTCTTGATCTGCCCAGT-3’ |
| IDH1_F888 | 5’- TTGTCCAGATGGCAAGACAG-3’ |
| IDH1_R1036 | 5’- GCTTTGCTCTGTGGGCTAAC-3’ |
| PGAM1_F585 | 5’- GGCTATCATGGAGCTGAACC-3’ |
| PGAM1_R710 | 5’- TCTTCATCCCCCAGAAACTG-3’ |
| TPI1_F573 | 5’- ATGGCTGAAGTCCAACGTCT-3’ |
| TPI1_R695 | 5’- ACAAGGAAGCCATCCACATC-3’ |
| HMGCS1_F1290 | 5’- AGAGGACACCCATCATTTGG-3’ |
| HMGCS1_R1405 | 5’- GCCGAGCGTAAGTTCTTCTG-3’ |
| IDI1_F601 | 5’- TGGGGTGAACATGAAATTGA-3’ |
| IDI1_R744 | 5’- AATTTCACCACTGGCTGCTT-3’ |
| NAMPT_F1305 | 5’- GCCAGCAGGGAATTTTGTTA-3’ |
| NAMPT_R1404 | 5’- TGTCACCTTGCCATTCTTGA-3’ |
| ITPA_F166 | 5’- AAATGTCAGGAGGCAGTTCG-3’ |
| ITPA_R309 | 5’- GAGCTGGTGGAGACCTTCAG-3’ |
| APRT_F190 | 5’- CTAGACTCCCGAGGCTTCCT-3’ |
| APRT_R336 | 5’- AATCTCCAGCTCAGCCTTCC-3’ |
| ADSS_F722 | 5’- CCCTACATGGACCACCAAAG-3’ |
| ADSS_R868 | 5’- CATTTTGAGGTGGCATACCC-3’ |
| PRPS1_F49 | 5’- GACATGGCTGACACTTGTGG-3’ |
| PRPS1_R154 | 5’- GACCGGAGAAGATTCCATGA-3’ |
| MTAP_F590 | 5’- CCACAGTTCCAGAGGTGGTT-3’ |
| MTAP_R695 | 5’- GAAACTGCTTCCTCGTGCTC-3’ |
| PSAT1_F282 | 5’- AGCAGGAAGGTGTGCTGACT-3’ |
| PSAT1_R387 | 5’- CCCAAGTTTAGGGTGAACGA-3’ |
| CBX3_F244 | 5’-GCTGGCAAAGAAAAAGATGG-3’ |
| CBX_R346 | 5’-CTCTTGGTTTGTCAGCAGCA-3’ |
| PIR_F482 | 5’- CCAAGGTTTACACTCGCACA-3’ |
| PIR_R621 | 5’- ATCATCGGGCCCAATATACA-3’ |
| EEF2_F612 | 5’- CCTCTATGCCAGTGTGCTGA-3’ |
| EEF2_R796 | 5’- TCCTGTTCAAAACCCCGTAG-3’ |
| eIF3H_F104 | 5’- CCGTGAAGCAAGTGCAGATA-3’ |
| eIF3H_R215 | 5’-ACAACCAGACCCAAAAGCAC-3’ |
| eI2B1_F117 | 5’- GGAGACAATCCAGGGTCTGA-3’ |
| eI2B1_R232 | 5’- GACTGATGAAGCGGAGGAAG-3’ |
| HNRNPC_F95 | 5’- ATGTGGAGGCAATCTTTTCG-3’ |
| HNRNPC_R218 | 5’- CTGCCATCCTCTCCTGCTAC-3’ |
| PCBP1_F96 | 5’- AGGGGAGTCGGTTAAGAGGA-3’ |
| PCBP1_R218 | 5’- TCTTCCTCCAGCTTGTCGAT-3’ |
| SSB_F90 | 5’- GCCACGGGACAAGTTTCTAA-3’ |
| SSB_R235 | 5’- GTTCTGCCTTGGATTTGCTC-3’ |
| KRT7_F508 | 5’- CAGGATGTGGTGGAGGACTT-3’ |
| KRT7_R623 | 5’- TTGCTCATGTAGGCAGCATC-3’ |
| ARPC2_F108 | 5’- AGATTTCGATGGGGTCCTCT-3’ |
| ARPC2_R212 | 5’- CCATGTGCCTGAAGTTCCTT-3’ |
| NPM1_F435 | 5’- TGGAGGTGGTAGCAAGGTTC-3’ |
| NPM1_R582 | 5’- TTTCTTCACTGGCGCTTTTT-3’ |
| CPNE1_F1394 | 5’- GACCCCTGCATACACGTTCT-3’ |
| CPNE1_R1542 | 5’- GGCCCTGAAGTATGAGACCA-3’ |
| GDI2_F965 | 5’- GACCAGCTTTGGAGCTCTTG-3’ |
| GDI2_R1072 | 5’- TGCGGGAAATAAAGATCTGG-3’ |
| ETFA_F224 | 5’- AAGTTCTGGTGGCTCAGCAT-3’ |
| EFTA_R373 | 5’- CTGCTACTCTGGGCAAAAGG-3’ |
| PPIA_F380 | 5’- TGGTGTTTGGCAAAGTGAAA-3’ |
| PPIA_R494 | 5’- TCGAGTTGTCCACAGTCAGC-3’ |
| RCN1_F202 | 5’- GACTCCAAGACCTTCGACCA-3’ |
| RCN1_R321 | 5’- CCAGGTTTTCAGCTCCTCAG-3’ |
| TXNDC17_F71 | 5’- GCAAGACCATTTTCGCCTAC-3’ |
| TXNDC17_R181 | 5’- TAATGTGCTTCAGCCCCTCT-3’ |

**^a^** The uppercases ‘F’ and ‘R’ indicate forward and reverse primer, respectively, whereas the numerals following the uppercase letter represent the position of the primer relative to the gene sequence.
